# From signal to significance: characterizing anthracycline-related hypercoagulable adverse events via pharmacovigilance, animal models, and clinical observation

**DOI:** 10.3389/fphar.2026.1856410

**Published:** 2026-07-31

**Authors:** Wen Huang, Junyi Shen, Jian Zhang

**Affiliations:** Department of Oncology, Zhujiang Hospital, Southern Medical University, Guangzhou, China

**Keywords:** anthracyclines, FAERS, hypercoagulability, pharmacovigilance, VigiBase

## Abstract

**Background:**

Anthracyclines are widely used antineoplastic agents with well-recognized cardiotoxicity; however, reports of hypercoagulable adverse events (HC-AEs) remain scattered and incompletely described. A systematic characterization of these events across large pharmacovigilance databases may help to clarify reporting patterns and generate hypotheses for further investigation.

**Methods:**

We performed a disproportionality analysis using the FDA Adverse Event Reporting System (FAERS) and the WHO Global Individual Case Safety Report Database (VigiBase) to identify HC-AEs reported in association with anthracyclines. Signals consistently observed in both databases were further explored descriptively with respect to reported drug-specific patterns and time-to-onset (TTO). To support hypothesis generation, exploratory analyses integrating transcriptomic data from The Cancer Genome Atlas (TCGA), retrospective clinical coagulation parameters, and *in vivo* observations from a tumor-bearing mouse model were conducted.

**Results:**

Across both pharmacovigilance platforms, 26 HC-AEs were consistently reported in association with anthracyclines. Doxorubicin was associated with the widest range of reported HC-AEs, whereas daunorubicin and aclarubicin showed more specific reporting patterns involving cerebral or intracardiac thrombosis and disseminated intravascular coagulation, respectively. The reported median TTO of HC-AEs following anthracycline exposure was 32.5 days (vs. 48.0 days in non-recipients, *P* < 0.001), with a predominance of early-onset events, particularly among younger patients (≤39 years). Exploratory analyses suggested potential involvement of pathways related to cyclic adenosine monophosphate (cAMP) signaling and heparan sulfate biosynthesis, which were directionally consistent with observed alterations in coagulation-related parameters in clinical samples and experimental models.

**Conclusion:**

This multi-database pharmacovigilance analysis provides a systematic overview of HC-AEs reported in association with anthracyclines, highlighting drug-specific reporting patterns and variations in onset timing across age groups. While causal inference cannot be established, the concordant signals observed across independent data sources support heightened clinical awareness and hypothesis-driven surveillance of thrombotic complications during anthracycline therapy.

## Introduction

Anthracyclines represent a critical class of antineoplastic agents, with key representatives including doxorubicin (DOX), epirubicin (EPI), and daunorubicin (DNR). Since their introduction into clinical practice in the 1950s, anthracyclines have demonstrated pivotal therapeutic efficacy in treating solid tumors and hematologic malignancies (Tuzovic et al., 2018; Mattioli et al., 2023). Clinical evidence has established anthracyclines as essential components of standard treatment protocols for numerous malignancies, including breast cancer, leukemia, lymphoma, and sarcoma (Lyon et al., 2022). The antineoplastic activity of anthracyclines operates through multiple molecular mechanisms, including programmed cell death induction, cellular necrosis, mitochondrial dysfunction, and modulation of key cellular pathways (Mattioli et al., 2023). Despite their notable cardiotoxicity, anthracyclines remain a cornerstone of modern cancer therapy due to their exceptional clinical efficacy. Recent advances in drug delivery technologies, particularly liposomal formulations and nanocarrier systems, have enhanced their targeting precision and safety profiles (Mattioli et al., 2023).

Hypercoagulable states are abnormal thrombotic tendencies resulting from inherited or acquired molecular defects (Thomas, 2001). These prothrombotic disorders can manifest as severe organ dysfunction and life-threatening complications. Cancer patients show an increased risk of hypercoagulability, particularly those with mucinous carcinoma, brain tumors, and acute promyelocytic leukemia (Thomas, 2001). Research has established hypercoagulability as the second leading cause of mortality in cancer patients (Abdol Razak et al., 2018). The primary manifestations include deep vein thrombosis (DVT) and arterial thrombosis (AT), affecting 15% of cancer patients clinically and up to 50% in post-mortem studies (Semchuk and Sperlich, 2012). Approximately 90% of cancer patients exhibit coagulation abnormalities, characterized by elevated levels of fibrinogen, coagulation factors, fibrin degradation products, and thrombocytosis (Hisada and Mackman, 2017).

Characterizing drug-associated hypercoagulability, particularly hypercoagulable events associated with anthracycline exposure, holds paramount clinical significance in chemotherapy. Prior preclinical and clinical studies suggest that anthracyclines may elevate thrombotic risk via platelet dysfunction and vascular endothelial injury (Wang et al., 2023; Grover et al., 2021). Clinical evidence suggests an association between anthracycline exposure and venous and arterial thrombotic events, including DVT, pulmonary embolism (PE), and AT. Notably, patients with breast cancer and multiple myeloma (MM) exhibit a significantly elevated risk (Nolan et al., 2011; Key et al., 2020). This risk peaks in patients aged >60 years and appears independent of traditional factors like obesity, diabetes, and hypertension (Goodnough et al., 1984; Sørensen et al., 2023). However, specific contributing patient factors remain incompletely understood. While potentially correlated with metabolic syndrome, tumor type, and protocols, direct causality warrants further investigation. Current research remains predominantly exploratory, with limited systematic investigation into clinical features and mechanisms.

Consequently, a comprehensive analysis elucidating the association between anthracyclines and hypercoagulable events, alongside their determinant factors, is critical for optimizing clinical management. This investigation utilized adverse event reports from the United States Food and Drug Administration (FDA) Adverse Event Reporting System (FAERS, Q1 2013–Q2 2025) and the World Health Organization’s Global Individual Case Safety Report Database (VigiBase, 1968–September 2025), employing disproportionality analysis to evaluate the characteristics of hypercoagulable events, while examining associated factors and underlying molecular mechanisms. This study aims to systematically characterize anthracycline-related hypercoagulable adverse events and generate pharmacovigilance signals and hypotheses to support future mechanistic and clinical investigation.

## Methods

### Data sources

This investigation utilized data from two comprehensive global pharmacovigilance databases: FAERS and VigiBase. FAERS serves as a publicly accessible repository integrating safety reports from patients, healthcare professionals, and pharmaceutical companies (Li et al., 2021), while VigiBase, established and operated by the Uppsala Monitoring Center, encompasses global adverse drug reaction reports (Lindquist, 2008). Using these databases, we performed a systematic pharmacovigilance analysis to evaluate coagulation-related adverse events associated with anthracyclines. The analysis encompassed anthracyclines and their derivatives, including doxorubicin, epirubicin, pirarubicin, daunorubicin, idarubicin, aclarubicin, and amrubicin, along with their corresponding trade names (Supplementary Table S1). The study period encompassed adverse reaction reports from January 1, 2013, to June 30, 2025, for FAERS, and from 1968 to September 2025, for VigiBase. Adverse reaction reports were classified using the preferred terms (PT) from the Medical Dictionary for Regulatory Activities (MedDRA version 25.1). PTs represent specific medical concepts—including signs, symptoms, and disease diagnoses—hierarchically organized into high-level terms (HLTs), higher-level group terms (HLGTs), and system organ classes (SOCs), based on etiology, manifestation site, or purpose. A comprehensive set of coagulation-related adverse reaction PTs (N = 628) was extracted from MedDRA for analysis (Supplementary Table S2). The analysis included only cases where anthracyclines were designated as “Primary Suspect (PS)” in FAERS or “suspect” in VigiBase. As these databases are publicly accessible and contain anonymized patient data, ethical approval and informed consent were not applicable for this study.

### Data processing

**FIGURE 1:**
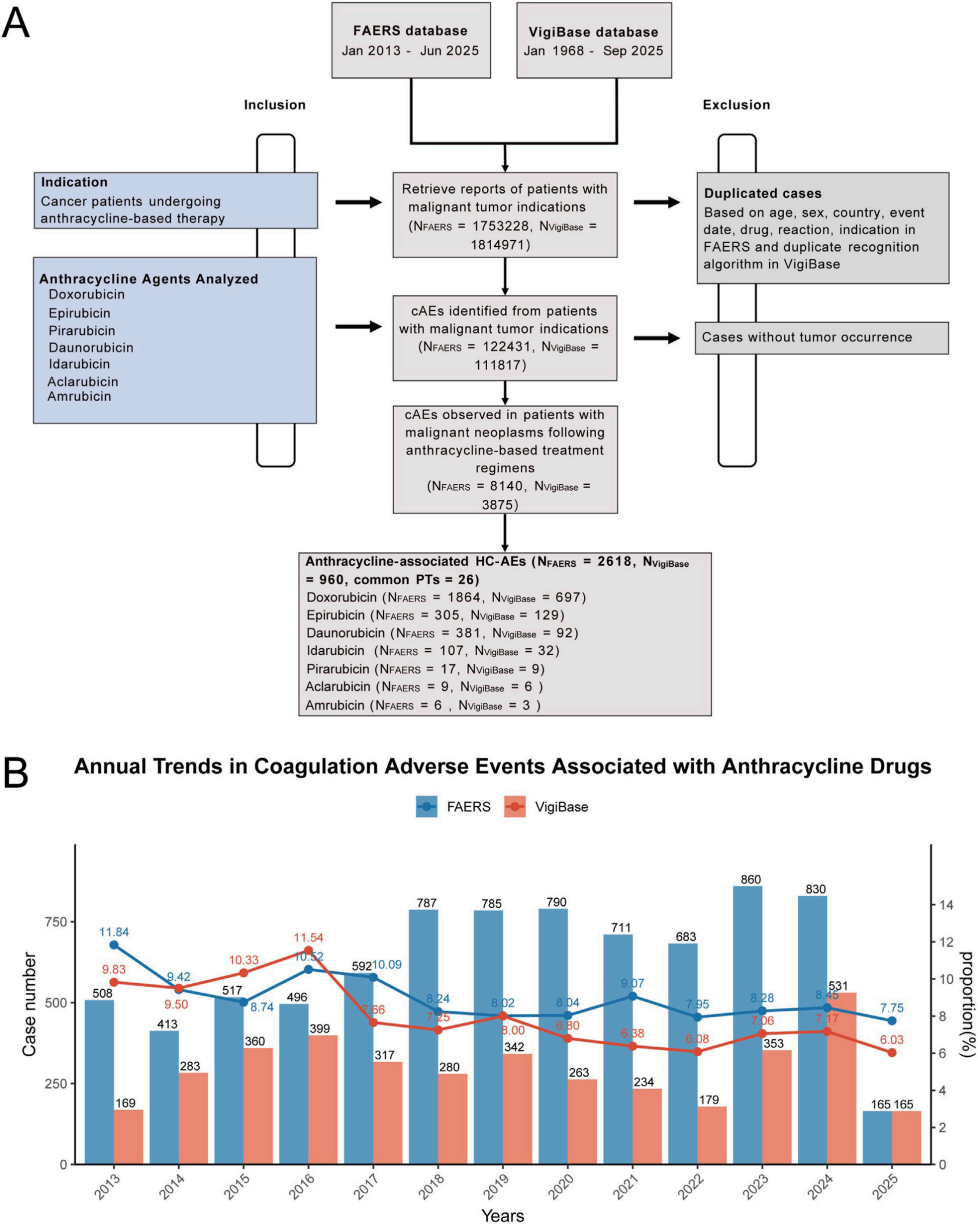
Identification and temporal reporting trends of anthracycline-associated coagulation-related adverse events. **(A)** Flowchart depicting the case selection process for coagulation-related AEs (cAEs) analysis from the United States Food and Drug Administration (FDA) Adverse Event Reporting System (FAERS) and the World Health Organization’s Global Individual Case Safety Report Database (VigiBase) databases. **(B)** Longitudinal trends in the reporting of anthracycline-related cAEs from 2013 to 2025. The dual-axis graph integrates data from both FAERS (blue) and VigiBase (red). The bar charts (left y-axis) represent the absolute annual frequency of reported cases, while the line graphs (right y-axis) depict the annual reporting proportion (%) of cAEs relative to all anthracycline-related adverse events. Abbreviations: cAEs, coagulation-related AEs; FAERS, United States Food and Drug Administration (FDA) Adverse Event Reporting System; VigiBase, World Health Organization’s Global Individual Case Safety Report Database.

A systematic analysis was conducted on coagulation-related adverse reactions associated with anthracyclines from the FAERS and VigiBase databases, with the detailed screening process illustrated in Figure 1A. Duplicate reports were identified using FDA-recommended methodology, wherein reports with identical sex, age, country, event date, adverse reaction, drug, and indication parameters were classified as duplicates (Khaleel et al., 2022). For VigiBase, duplicate elimination was performed using a standardized, proprietary algorithm (Gastaldon et al., 2023). Following de-duplication, analysis was restricted to adverse reaction reports specifically associated with malignancy indications (Supplementary Table S3). To evaluate temporal trends, we calculated the annual reporting proportion, defined as the proportion of coagulation-related AEs (cAEs) among all adverse events (AEs) reported for anthracyclines annually in each database.

### Signal mining

We employed disproportionality analysis, a fundamental pharmacovigilance method, to evaluate drug-AE associations (Caster et al., 2020). Specifically, reporting odds ratios (RORs) were calculated to quantify associations (Sakaeda et al., 2011). A 2 × 2 contingency table was constructed to analyze anthracycline-related cAEs (Supplementary Table S4). Parameters included: (a) number of reports listing anthracyclines with cAEs; (b) number of reports listing anthracyclines without cAEs; (c) number of reports listing other drugs with cAEs; and (d) number of reports listing neither anthracyclines nor cAEs. RORs and 95% confidence intervals (CIs) were computed as follows:

$$ROR=\frac{a^{*}\;d}{c^{*}\;b}$$

$$95\%\text{CI}=e^{\mathrm{Ln}\left(\text{ROR}\right)\pm 1.96\sqrt{\frac{1}{a}+\frac{1}{b}+\frac{1}{c}+\frac{1}{d}}}$$

Signal detection criteria included N ≥ 3 (total reports) and a 95% CI lower bound of the ROR >1 (Yan et al., 2022). Coagulation disorder PTs fulfilling these criteria were designated as anthracycline-related cAEs. Analysis revealed that hypercoagulable state-associated PTs constituted a significant proportion of anthracycline-related cAEs. To ensure robust analysis, overlapping reports between FAERS and VigiBase were classified as anthracycline-related hypercoagulable adverse events (HC-AEs) and utilized for subsequent detailed investigation. It should be emphasized that the ROR is a disproportionality measure reflecting the relative frequency with which an adverse event is reported for anthracyclines compared with all other drugs in the database; it does not represent an incidence rate, absolute risk, or a causal estimate. Accordingly, the term ‘HC-AEs’ as used throughout this manuscript denotes a pharmacovigilance signal—i.e., a pattern of disproportionate reporting—rather than a confirmed drug-induced or causally attributable clinical event.

### Subgroup analysis

A comprehensive subgroup analysis was performed to evaluate clinical characteristics of anthracycline-related HC-AEs, stratified by primary tumor system origin. The analysis encompassed tumors of the integumentary, endocrine, gastrointestinal, hematolymphoid, hepatobiliary, musculoskeletal, nervous, reproductive, respiratory, and urinary systems. Signal mining analyses were conducted independently for each stratified population subgroup. Furthermore, separate ROR analyses were conducted for individual anthracycline agents.

### Time-to-onset analysis

We calculated time-to-onset (TTO) as the interval between medication initiation (START_DT) and adverse event occurrence (EVENT_DT) in FAERS. Reports with missing or erroneous dates were excluded; however, for records with only year and month available, we imputed the date as the first day of the corresponding month (Yan et al., 2025). Overall, 12,306 reports were included in the primary TTO analysis (2,331 with imputed dates; 9,975 with precise dates). A sensitivity analysis was conducted using only the 9,975 precise-date reports. Temporal patterns stratified by sex, age, and drug type were evaluated using cumulative distribution curves and Kaplan-Meier analyses.

### Establishment of the tumor-bearing mouse model and anthracycline treatment protocol

All animal experiments were conducted under protocols approved by the Experimental Animal Ethics Committee of Zhujiang Hospital, Southern Medical University (LAEC-2023–222), and complied with the International Guidelines for Laboratory Animal Use. For all studies, 6-8-week-old male C57BL/6 mice were purchased and used from Shanghai Jihui Laboratory Animal Care. B16-F10 murine melanoma cells were maintained in Dulbecco’s Modified Eagle Medium (DMEM) supplemented with 10% fetal bovine serum and 1% penicillin-streptomycin at 37 °C in a humidified 5% CO2 atmosphere. Cells in logarithmic growth phase were harvested, resuspended in phosphate-buffered saline (PBS), and adjusted to 1 × 10^6^ cells/100 μL (viability >95%). Mice were housed under specific pathogen-free (SPF) conditions and inoculated subcutaneously with 100 μL cell suspension in the right flank. Following successful tumor establishment, tumor-bearing mice were randomly allocated into two groups (n = 6/group; doxorubicin vs. PBS). Body weight and clinical signs were monitored throughout the 4-week observation period. Humane endpoints were predefined in accordance with the approved ethics protocol (LAEC-2023–222) and included: >20% body weight loss from baseline, subcutaneous tumor diameter exceeding 20 mm, inability to feed or drink, respiratory distress, or moribund state. Outcome assessors responsible for body-weight monitoring, tumor measurement, coagulation testing, and statistical analysis were blinded to group allocation; samples were labeled with coded identifiers, and group information was revealed only after completion of data analysis. Coagulation parameters were collected at a single pre-specified terminal time point (week 4) across all animals. Treatments were administered via intraperitoneal (i.p.) injection. The treatment group received doxorubicin (2.5 mg/kg, twice weekly) for 4 weeks, while control mice received an equivalent volume of PBS as a vehicle control. All agents were purchased from Selleck Chemicals. Following the 4-week treatment period, mice were euthanized via intraperitoneal injection of an overdose of sodium pentobarbital. Immediately following euthanasia, peripheral blood was collected via terminal cardiac puncture. A portion of the sample was used for the analysis of coagulation parameters, including prothrombin time (PT), activated partial thromboplastin time (APTT), plateletcrit (PCT), platelet count (PLT), platelet distribution width (PDW), and fibrinogen (FIB), using standardized assays. Relevant tissue specimens were immediately snap-frozen in liquid nitrogen and stored at −80 °C for RNA extraction and subsequent high-throughput transcriptomic sequencing.

### Transcriptomic and pathway enrichment analyses

We accessed transcriptomic data in fragments per kilobase of transcript per million fragments mapped (FPKM) format for multiple cancer types from The Cancer Genome Atlas (TCGA) cohort through the University of California, Santa Cruz (UCSC) Xena database (Goldman et al., 2020), and converted them to transcripts per million (TPM) values for subsequent analysis (Wagner et al., 2012). Pan-cancer single-sample gene set enrichment analysis (ssGSEA) was performed using the gene set variation analysis (GSVA) software package (Hänzelmann et al., 2013). Comprehensive pathway analysis was conducted using Gene Ontology (GO), Kyoto Encyclopedia of Genes and Genomes (KEGG), and Reactome pathways from the Molecular Signatures Database (MSigDB), with enrichment scores calculated across cancer types (Subramanian et al., 2005). Enrichment scores quantify the collective up- or downregulation of gene set members within specific biological processes (Barbie et al., 2009). Pathway activation levels in specific cancer types were represented by mean enrichment scores (Jing et al., 2020). Correlations between anthracycline-related HC-AE RORs and pathway activation levels were analyzed across cancer types to elucidate potential molecular mechanisms.

For the *in vivo* mechanistic investigation, Gene Set Enrichment Analysis (GSEA) was employed to identify anthracycline-responsive biological pathways. Transcriptional changes were evaluated in both our in-house tumor-bearing mouse model and publicly available rat data from the Gene Expression Omnibus (GEO) dataset (GSE37260) (Todorova et al., 2012). For the mouse model, total RNA was extracted from the snap-frozen specimens using TRIzol reagent, and quality-verified cDNA libraries were constructed. Sequencing data were aligned to the mouse reference genome (mm39) using HISAT2. Gene expression quantification was performed using htseq-count, followed by data normalization and differential expression analysis using DESeq2. Differential expression analysis compared anthracycline-treated groups (doxorubicin-treated samples GSM915058–GSM915066 for rat; doxorubicin-treated samples for mouse) against their respective vehicle controls (GSM915050–GSM915057 for rat; PBS for mouse). Log_2_ fold change (log_2_FC) values quantified differential expression, followed by rigorous quality control, including removal of duplicate genes and non-finite values. Filtered genes were ranked by log_2_FC magnitude to generate named vectors. Enrichment analysis was performed using the fgsea package, with statistical significance defined as an adjusted *P*-value (FDR) < 0.05. Normalized enrichment scores (NES) determined pathway activation status. Visualization of GSEA results was facilitated by the GseaVis package (Zhang et al., 2025).

### Clinical observation of coagulation parameters

We conducted a retrospective observational study to evaluate coagulation dynamics in patients receiving anthracycline-based chemotherapy at the Department of Oncology, Zhujiang Hospital of Southern Medical University. The study period spanned from 2016 to 2024, during which eligible patients were identified from the hospital’s electronic medical record system. The inclusion criteria were defined as: (1) pathologically or clinically confirmed malignant tumor; (2) receipt of at least one anthracycline agent, including doxorubicin, epirubicin, pirarubicin, daunorubicin, or idarubicin; and (3) availability of coagulation-related laboratory measurements, specifically prothrombin activity (PTA) or antithrombin III (AT-III), obtained both before (baseline) and after anthracycline administration. Patients were strictly excluded if they met any of the following criteria: (1) incomplete laboratory records or protocol interruptions; (2) pre-existing severe coagulopathy or hepatic dysfunction at baseline (3) concurrent administration of anticoagulants (e.g., heparin, low-molecular-weight heparin, direct oral anticoagulants, or warfarin); or antiplatelet agents during the observation period; and (4) acute infections or other conditions profoundly confounding coagulation. Following these selection criteria, a total of 572 patients were included for PTA analysis, and 482 patients were included for AT-III analysis. Coagulation function was classified into three clinical categories based on standardized reference ranges: hypercoagulable (PTA >100%, AT-III <80%), hypocoagulable (PTA <75%, AT-III >120%), and normal (PTA 75%–100%, AT-III 80%–120%). The cohort included patients with hematological malignancies and solid tumors; post-treatment samples were obtained within the same chemotherapy cycle as anthracycline administration during routine clinical monitoring. This study was approved by the Ethics Committee of Zhujiang Hospital of Southern Medical University (Approval ID: 2024-KY-129–01), and all procedures were performed in accordance with the Declaration of Helsinki.

### Statistical analysis

Categorical and continuous variables were compared using Chi-square tests and paired or independent t-tests, respectively. Linear mixed-effects models evaluated temporal trends. TTO distributions were analyzed using Kaplan-Meier curves and log-rank tests, while median TTO differences were assessed via Mann-Whitney U and Kruskal–Wallis tests. Spearman’s rank correlation evaluated relationships between pan-cancer HC-AE RORs and pathway enrichment. Results were visualized using heatmaps, forest plots, and histograms. Analyses utilized R software (v4.2.0) and ggplot2. To control for multiple testing, false discovery rate (FDR) values were calculated using the Benjamini–Hochberg method via the p. adjust function, with significance set at two-sided FDR or *P* < 0.05.

## Results

### Coagulation-related adverse events among users of anthracyclines in FAERS and VigiBase, 2013–2025

Our analysis identified 8,140 anthracycline-related cAEs from 1,753,228 FAERS reports and 3,875 anthracycline-related cAEs from 1,814,971 VigiBase reports, ultimately forming the analysis dataset (Figure 1A). Temporal analysis of both FAERS and VigiBase databases revealed annual trends in anthracycline-related cAE reporting from 2013 to 2025 (Figure 1B). Analysis of the FAERS database demonstrated a progressive annual increase in anthracycline-related cAE reports, reaching a peak of 860 cases in 2023. However, its proportion among all anthracycline-related adverse events peaked at 11.84% in 2013, subsequently stabilizing at approximately 8% after 2018. Similar trends were observed in the VigiBase database, with anthracycline-related cAE reports increasing significantly to 531 cases in 2024; however, their proportion peaked at 11.54% in 2016 and has gradually declined since, falling to 6.03% by 2025.

### Overview of anthracycline-related HC-AEs

**FIGURE 2:**
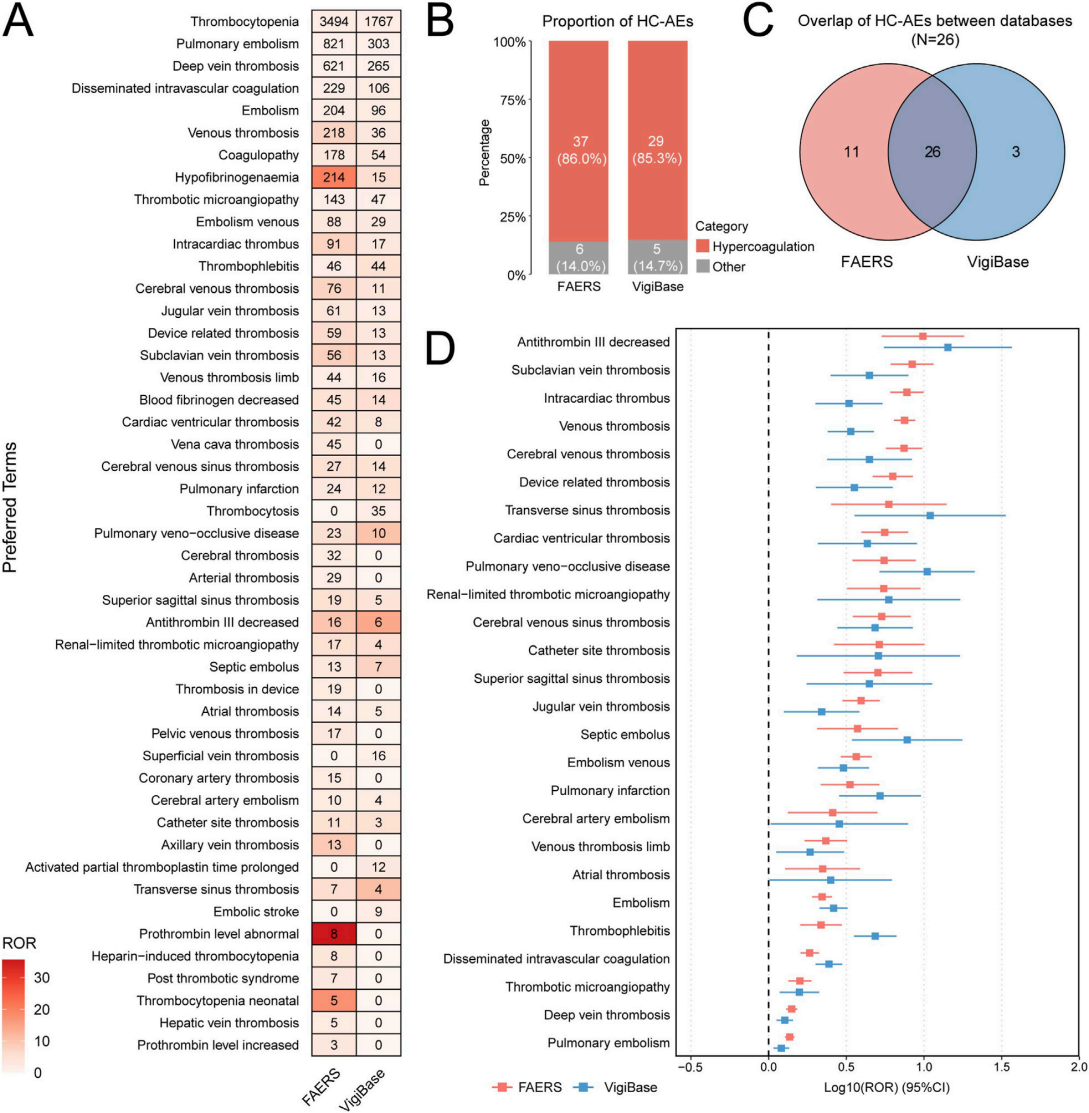
Spectrum and disproportionality signals of anthracycline-related cAEs. **(A)** Heatmap illustrating both signal strength and reporting frequency of positive signal cAEs identified in FAERS and VigiBase. The color gradient represents the signal strength (reporting odds ratio, ROR), with darker red indicating a stronger drug-event association. The numerical values within each cell represent the absolute reporting frequency (case counts). **(B)** Classification and proportion of identified anthracycline-related cAEs in FAERS and VigiBase. The stacked bar charts display the distribution of Preferred Terms (PTs) categorized as “Hypercoagulation” (red) versus “Other” (grey). The values inside the bars represent the absolute number of PTs (e.g., 37 and 29) and their respective percentages. Hypercoagulation includes thrombotic and prothrombotic events (e.g., venous thrombosis, embolism), whereas “Other” refers to coagulation-related abnormalities not directly thrombotic (e.g., thrombocytopenia, hypofibrinogenemia). **(C)** Venn diagram demonstrating the overlap of anthracycline-related hypercoagulable adverse events (HC-AEs) reported in FAERS (N = 37) and VigiBase (N = 29), revealing 26 PTs shared between the two databases. **(D)** Forest plot comparing the Log10-transformed RORs and 95% confidence intervals for anthracycline-related HC-AEs jointly reported across FAERS and VigiBase. Abbreviations: cAEs, coagulation-related AEs; FAERS, Food and Drug Administration Adverse Event Reporting System; HC-AEs, hypercoagulable adverse events; PT, preferred term; ROR, reporting odds ratio; VigiBase, WHO Global Database of Individual Case Safety Reports.

Analysis identified 43 and 34 target PTs from the FAERS and VigiBase databases, respectively. Within the broad category of initial cAEs, thrombocytopenia emerged as the most frequent adverse event (N_FAERS_ = 3494; N_VigiBase_ = 1767), followed by pulmonary embolism (N_FAERS_ = 821; N_VigiBase_ = 303) and deep vein thrombosis (N_FAERS_ = 621; N_VigiBase_ = 265) (Figure 2A). Hypercoagulation-related events constituted the majority of targeted adverse events (FAERS: 37/43; VigiBase: 29/34) (Figure 2B). Cross-database analysis identified 26 common PTs, subsequently designated as anthracycline-related HC-AEs (Figure 2C). When stratified by ROR magnitude, the most significant signals encompassed antithrombin III decreased (ROR_FAERS_ = 9.83, 95% CI: 5.34–18.11; ROR_VigiBase_ = 14.26, 95% CI: 5.53–36.75), subclavian vein thrombosis (ROR_FAERS_ = 8.39, 95% CI: 6.11–11.53; ROR_VigiBase_ = 4.46, 95% CI: 2.50–7.93), intracardiac thrombus (ROR_FAERS_ = 7.77, 95% CI: 6.07–9.94; ROR_VigiBase_ = 3.29, 95% CI: 2.00–5.41), and venous thrombosis (ROR_FAERS_ = 7.50, 95% CI: 6.40–8.79; ROR_VigiBase_ = 3.38, 95% CI: 2.40–4.75) (Figure 2D). Sensitivity analysis restricted to clinically overt thrombotic events yielded directionally consistent results, supporting the robustness of the HC-AE signal definition (Supplementary Table S5).

### Subgroup analysis of anthracycline-related HC-AEs

Drug-specific subgroup analysis revealed distinct coagulation toxicity profiles across different anthracycline drugs (Supplementary Figure 1). First, in terms of the breadth of positive signal coverage, doxorubicin exhibited the most extensive toxicity spectrum across both databases, correlating with multiple positive PTs (FAERS: 23/26; VigiBase: 20/26). This was followed by daunorubicin (FAERS: 16/26; VigiBase: 6/26) and epirubicin (FAERS: 12/26; VigiBase: 7/26), suggesting that these drugs may be associated with disproportionate reporting of multiple types of coagulation complications. Further analysis of PTs commonly associated with these high-frequency drugs revealed disseminated intravascular coagulation as a frequently occurring signal, particularly significant for aclarubicin (ROR_VigiBase_ = 82.62, 95% CI: 33.29–205.08) and daunorubicin (ROR_VigiBase_ = 8.62, 95% CI: 5.54–13.42). Beyond broad-spectrum toxicities, we identified several drug-specific signals with robust correlations and ROR values significantly higher than other combinations, warranting close attention. For specific anthracyclines, daunorubicin demonstrated exceptionally strong associations with cerebral venous thrombosis (ROR_FAERS_ = 69.86, 95% CI: 52.23–93.43; ROR_VigiBase_ = 60.40, 95% CI: 29.26–124.68), intracardiac thrombus (ROR_FAERS_ = 51.80, 95% CI: 38.20–70.24; ROR_VigiBase_ = 25.27, 95% CI: 11.87–53.79), and cerebral venous sinus thrombosis (ROR_FAERS_ = 32.23, 95% CI: 18.35–56.63; ROR_VigiBase_ = 32.22, 95% CI: 13.12–79.11). Notably, doxorubicin showed robust signals for antithrombin III decreased (ROR_FAERS_ = 9.95, 95% CI: 5.08–19.50; ROR_VigiBase_ = 12.10, 95% CI: 4.01–36.45) and pulmonary veno-occlusive disease (ROR_FAERS_ = 5.20, 95% CI: 3.03–8.93; ROR_VigiBase_ = 10.67, 95% CI: 4.94–23.06). Additionally, epirubicin presented a strong correlation with thrombophlebitis in VigiBase (ROR = 18.01, 95% CI: 11.46–28.31).

Subgroup analysis of the FAERS database was conducted to evaluate the relationship between tumor site and the reporting pattern of anthracycline-associated hypercoagulability signals. HC-AEs predominantly occurred in patients with hematological malignancies (N = 2,064) and breast cancer (N = 419). In hematological malignancies, pulmonary embolism (N = 518) and deep vein thrombosis (N = 473) were the most frequent events, while venous thrombosis (ROR = 12.99, 95% CI: 10.63–15.88), subclavian vein thrombosis (ROR = 11.51, 95% CI: 7.17–18.46), cerebral venous thrombosis (ROR = 9.88, 95% CI: 7.22–13.51), and cardiac ventricular thrombosis (ROR = 9.30, 95% CI: 5.21–16.58) exhibited elevated ROR values. In contrast, breast cancer cases showed significant signals for subclavian vein thrombosis (ROR = 5.23, 95% CI: 2.89–9.45), cerebral artery embolism (ROR = 4.77, 95% CI: 1.35–16.90), and intracardiac thrombus (ROR = 4.65, 95% CI: 2.31–9.35), although pulmonary embolism (N = 145) and deep vein thrombosis (N = 70) remained the most frequently reported events (Supplementary Figure 2).

### TTO analysis of anthracycline-related HC-AEs

**FIGURE 3:**
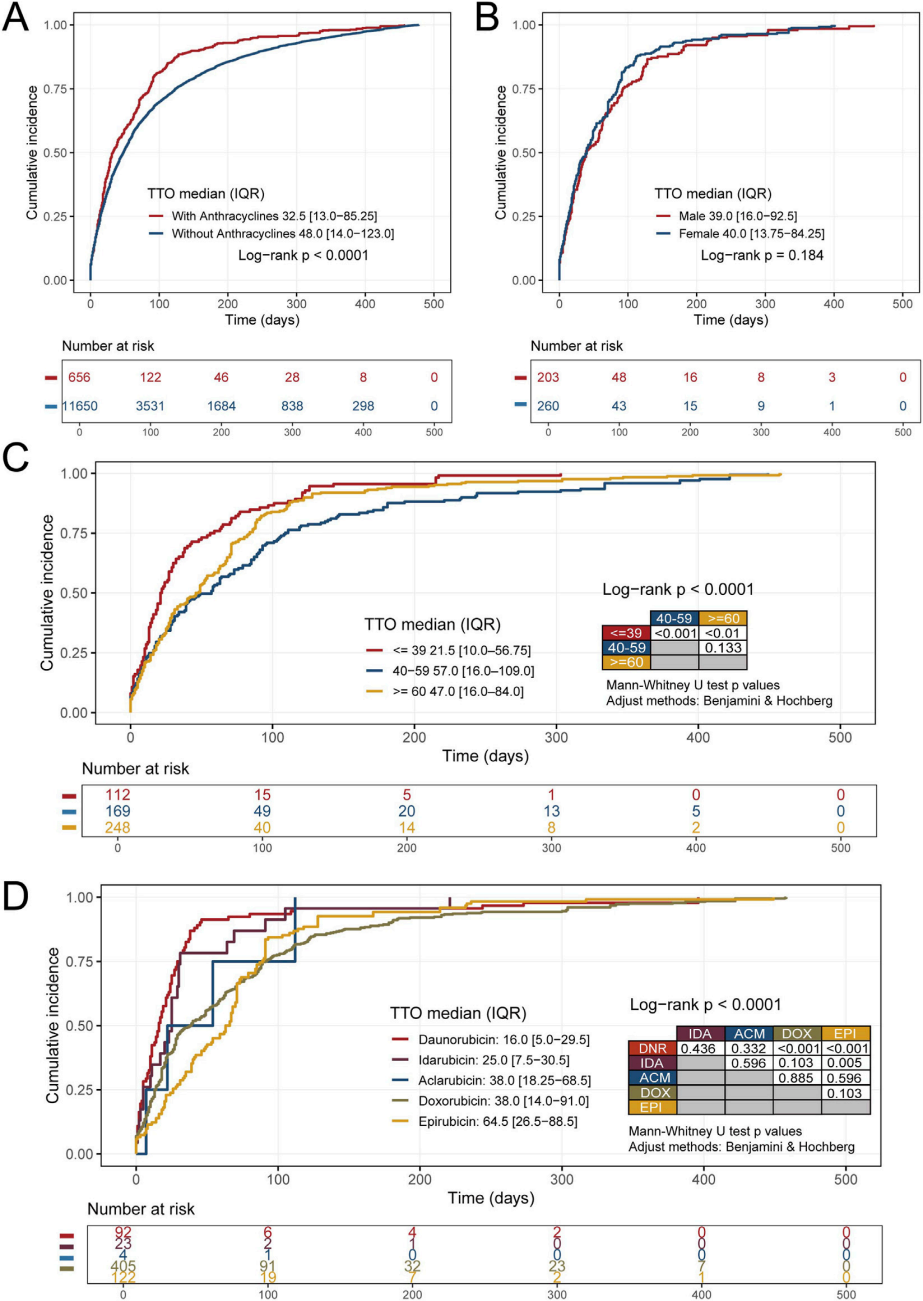
Time-to-onset analysis of HC-AEs. **(A)** Kaplan-Meier curve comparing the time-to-onset of HC-AEs between patients with and without anthracycline exposure. **(B)** Comparison of the time-to-onset (TTO) of HC-AEs stratified by sex (Female vs. Male). **(C)** Analysis of the TTO of HC-AEs stratified by age group (≤39, 40–59, and ≥60 years). The inset table displays adjusted P-values for pairwise comparisons between age groups. **(D)** Analysis of the TTO of HC-AEs stratified by individual anthracycline agents (Daunorubicin, Idarubicin, Aclarubicin, Doxorubicin, and Epirubicin). The inset table provides the matrix of adjusted P-values for pairwise comparisons between specific drugs. For all panels, the number of patients at risk and median TTO with interquartile range (IQR) are provided below the curves or in the legend. Statistical analyses employed the Mann–Whitney U test, and pairwise comparisons were conducted using the Kruskal–Wallis test, with corrections applied using the Benjamini and Hochberg method. Abbreviations: ACM, aclarubicin; DNR, daunorubicin; DOX, doxorubicin; EPI, epirubicin; HC-AEs, hypercoagulable adverse events; IDA, idarubicin; IQR, interquartile range; TTO, time-to-onset.

To characterize the temporal patterns of anthracycline-related HC-AEs, we performed a TTO analysis. TTO analysis demonstrated significantly shorter onset times in the anthracycline-exposed group compared to the non-exposed group (median TTO: 32.5 [IQR: 13.0–85.3] vs. 48.0 [14.0–123.0] days; *P* < 0.001) (Figure 3A). Sex-stratified analysis revealed no significant difference in median TTO between male and female patients (39.0 [16.0–92.5] vs. 40.0 [13.8–84.3] days; *P* = 0.184) (Figure 3B). Age-stratified analysis revealed significant differences in median TTO across age groups (*P* < 0.001) (Figure 3C). Pairwise comparisons indicated that the median TTO was significantly shorter in patients aged ≤39 years compared to those aged 40–59 years (≤39 years: 21.5 [10.0–56.8] vs. 40–59 years: 57.0 [16.0–109.0] days; *P* < 0.001) and ≥60 years (47.0 [16.0–84.0] days; *P* < 0.01). Drug-specific analysis demonstrated significant heterogeneity in median TTO across anthracycline drugs (*P* < 0.001). Specifically, daunorubicin exhibited the shortest median TTO (16.0 [5.0–29.5] days), while epirubicin had the longest (64.5 [26.5–88.5] days) (Figure 3D). Pairwise comparisons revealed that the median TTO for daunorubicin was significantly shorter than that of doxorubicin (38.0 [14.0–91.0] days; *P* < 0.001) and epirubicin (*P* < 0.001). A sensitivity analysis excluding the 2,331 imputed reports yielded highly consistent TTO results across all subgroups, confirming the robustness of these findings (Supplementary Table S6). These findings suggest that reported HC-AE onset may occur earlier in anthracycline-exposed patients, with timing patterns that vary by age and drug class.

### Analysis of the intrinsic biological mechanisms associated with anthracycline-related HC-AEs

**FIGURE 4:**
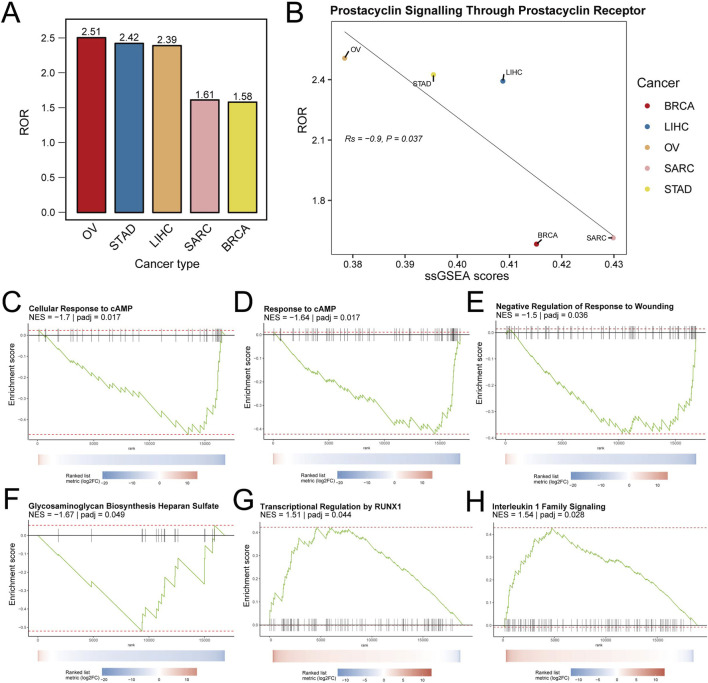
Cancer-specific variations in thrombotic risk and associated signaling pathways. **(A,B)** Evaluation of clinical thrombotic risk and molecular correlations in human cancers. **(A)** Bar plot depicting the RORs for HC-AEs across five cancer types. Cancer types with fewer than three reported cases were excluded from the analysis. **(B)** Scatter plot illustrating the negative association (R = −0.9, *FDR* = 0.037) between ROR values and single-sample gene set enrichment analysis (ssGSEA) enrichment scores for the “Prostacyclin Signalling Through Prostacyclin Receptor” pathway. The Spearman’s rank correlation coefficient was used for correlation analysis. **(C–H)** Exploratory pathway analysis of anthracycline-associated transcriptomic alterations. **(C–F)** Gene set enrichment analysis (GSEA) from the in-house tumor-bearing mouse model (Doxorubicin vs. PBS, n = 6 per group). Plots show pathways with negative Normalized Enrichment Scores (NES), confirming downregulation of: **(C)** Cellular Response to cyclic adenosine monophosphate (cAMP), **(D)** Response to cAMP, **(E)** Negative Regulation of Response to Wounding, and **(F)** Glycosaminoglycan Biosynthesis–Heparan Sulfate. **(G,H)** GSEA validation from an independent rat transcriptomic dataset (GSE37260). Plots show pathways with positive NES, confirming upregulation of: **(G)** Transcriptional Regulation by RUNX1 and **(H)** Interleukin-1 Family Signaling. All GSEA pathways shown satisfy statistical significance (*FDR* < 0.05). Abbreviations: BRCA, Breast invasive carcinoma; cAMP, cyclic adenosine monophosphate; GSEA, gene set enrichment analysis; HC-AEs, hypercoagulable adverse events; LIHC, Liver hepatocellular carcinoma; NES, normalized enrichment score; OV, Ovarian serous cystadenocarcinoma; ROR, reporting odds ratio; SARC, Sarcoma; ssGSEA, single-sample gene set enrichment analysis; STAD, Stomach adenocarcinoma; TCGA, The Cancer Genome Atlas.

Initial analysis revealed differential signal strength of HC-AEs across malignancies, with ovarian cancer (OV) exhibiting the highest ROR score (Figure 4A). Subsequent pan-cancer transcriptome analysis of TCGA data identified a preliminary exploratory negative correlation between anthracycline-associated HC-AE reporting odds and prostacyclin receptor signaling (*R* = −0.90, *FDR* = 0.037). This exploratory correlation raises the hypothesis that altered prostacyclin receptor signaling may be mechanistically relevant to the hypercoagulability signals associated with anthracycline exposure (Figure 4B). GSEA identified anthracycline-related signaling pathways across *in vivo* rodent models, with mouse experiments revealing significant enrichment (*FDR* < 0.05) in pathways including Cellular Response to Cyclic Adenosine Monophosphate (cAMP) (NES = −1.70, *FDR* = 0.017) (Figure 4C), Response to cAMP (NES = −1.64, *FDR* = 0.017) (Figure 4D), Negative Regulation of Response to Wounding (NES = −1.50, *FDR* = 0.036) (Figure 4E), and Glycosaminoglycan Biosynthesis Heparan Sulfate (NES = −1.67, *FDR* = 0.049) (Figure 4F); these pathways—implicating cAMP signaling dysregulation, suppression of wound repair, and heparan sulfate synthesis—may represent plausible mechanistic contributors to the hypercoagulability signals associated with anthracycline exposure. Complementary GSEA of rat transcriptomic data (GEO: GSE37260) further identified significant enrichment (*FDR* < 0.05) in Transcriptional Regulation by RUNX1 (NES = 1.51, *FDR* = 0.044) (Figure 4G) and Interleukin-1 (IL-1) Family Signaling (NES = 1.54, *FDR* = 0.028) (Figure 4H), suggesting potential involvement of IL-1 signaling and RUNX1-mediated transcriptional programs in anthracycline response mechanisms.

### Integrated clinical and preclinical observations on anthracycline-associated hypercoagulability

**FIGURE 5:**
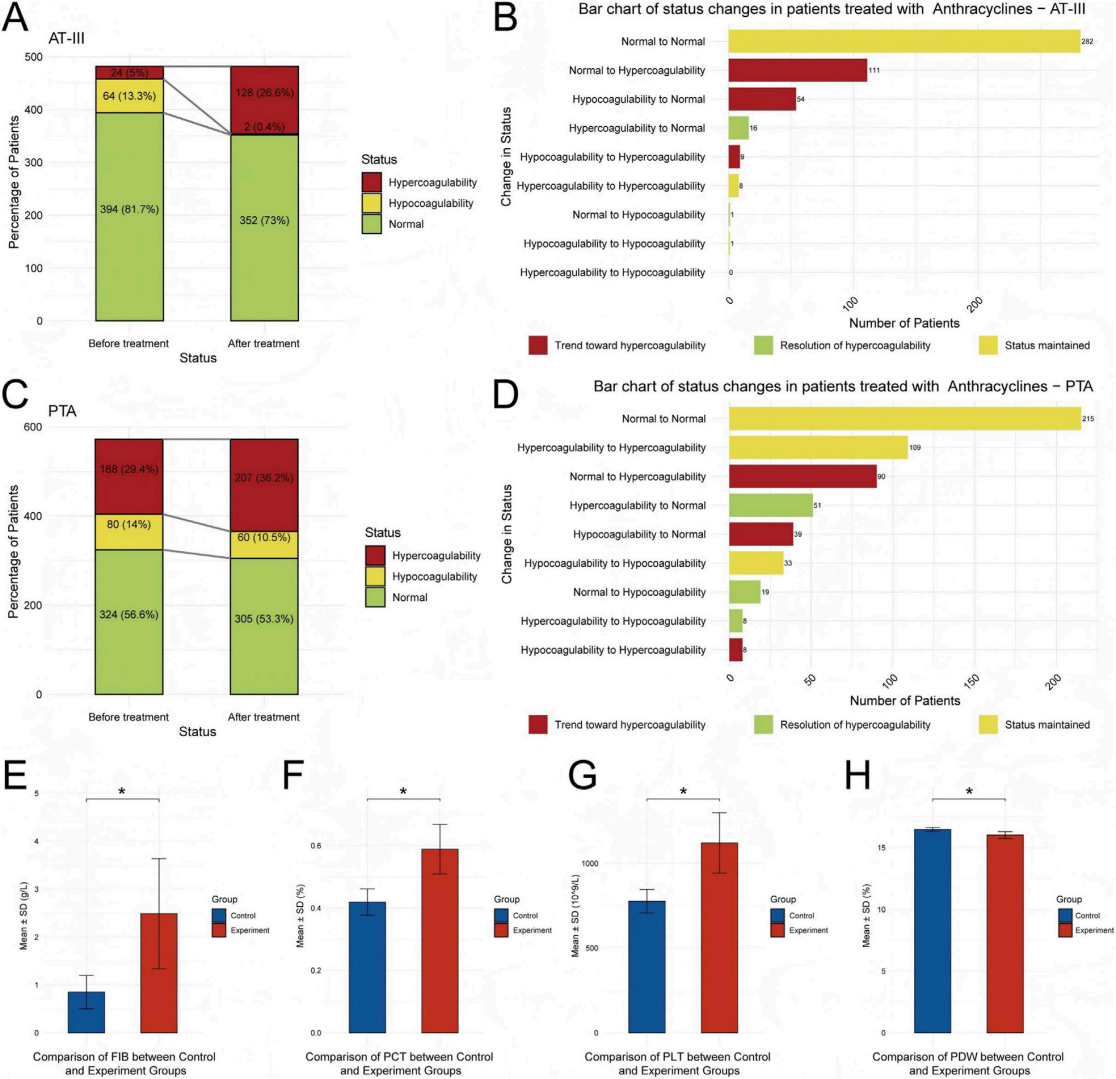
Coagulation status transitions and hematological alterations associated with anthracycline treatment. **(A–D)** Retrospective analysis of coagulation status in cancer patients. **(A,C)** Stacked bar charts illustrating the distribution of coagulation status (Normal, Hypocoagulability, Hypercoagulability) before and after anthracycline treatment based on **(A)** Antithrombin III (AT-III, N = 482) and **(C)** Prothrombin activity (PTA, N = 572). **(B,D)** Change in coagulation status for individual patients, categorized as “Trend toward hypercoagulability” (red), “Resolution of hypercoagulability” (green), and “Status maintained” (yellow) for **(B)** AT-III and **(D)** PTA. **(E–H)** Experimental validation of hematological alterations in tumor-bearing mice. Bar graphs comparing coagulation parameters between phosphate-buffered saline (PBS)-treated controls (blue) and Doxorubicin-treated mice (red, Experiment, n = 6 per group). Data are presented as mean ± standard deviation (SD). **(E)** Fibrinogen (FIB) levels; **(F)** Plateletcrit (PCT); **(G)** Platelet count (PLT); and **(H)** Platelet distribution width (PDW). Statistical significance is indicated by *P < 0.05. Abbreviations: AT-III, antithrombin III; FIB, fibrinogen; PCT, plateletcrit; PDW, platelet distribution width; PLT, platelet count; PTA, prothrombin activity; SD, standard deviation.

Retrospective clinical data analysis revealed laboratory trends consistent with hypercoagulability following anthracycline administration. Paired t-test analysis demonstrated significant alterations in both absolute coagulation indices (P < 0.001). Specifically, mean AT-III levels decreased from 105.56 at baseline to 88.39 post-treatment, while mean PTA levels increased from 91.41 to 94.17. Categorical analysis of clinical states showed a significant increase in hypercoagulable cases and a concurrent decrease in normocoagulable and hypocoagulable states following drug administration (Figures 5A,C). Chi-square analysis confirmed significant state transitions for both indices (AT-III: *P* < 0.001; PTA: *P* < 0.05). Detailed analysis of nine possible state transitions was performed (Figures 5B,D). Linear mixed-effects modeling revealed a gradual decrease in AT-III activity and an increasing trend in PTA over time, further supporting these exploratory observations.

In our preclinical studies, anthracycline treatment induced significant alterations in several key coagulation parameters when compared with the control group. FIB levels exhibited a significant elevation in the anthracycline-treated group (2.49 vs. 0.85, *P* = 0.030) (Figure 5E). Similarly, PCT levels demonstrated a pronounced increase in the anthracycline-treated group (0.59 vs. 0.42, *P* = 0.015) (Figure 5F). Furthermore, PLT was substantially higher in the anthracycline-treated group (1,120.50 vs. 777.25 × 10^9^/L, *P* = 0.024) (Figure 5G). In contrast, PDW exhibited a statistically significant, albeit slight, reduction in the anthracycline-treated group (16.03 vs. 16.48, *P* = 0.038) (Figure 5H). These findings collectively indicate alterations in platelet-related parameters and elevated fibrinogen levels following anthracycline exposure.

## Discussion

Although the association between anthracyclines and HC-AEs is recognized, a comprehensive characterization remains lacking (Kim et al., 2011). This study presents the first large-scale pharmacovigilance analysis using FAERS and VigiBase to systematically evaluate these events. Disproportionality analysis identified a broad spectrum of disproportionately reported coagulation events, ranging from frequent PE and DVT to severe, life-threatening signals such as cerebral venous thrombosis, intracardiac thrombus, and pulmonary veno-occlusive disease. Furthermore, longitudinal analysis demonstrated a progressive rise in reporting burden, underscoring the growing clinical relevance of these abnormalities. Integrating pan-cancer transcriptomic data revealed an exploratory negative correlation between anthracycline-associated HC-AE reporting odds and prostacyclin receptor signaling, suggesting a potential mechanism for hypercoagulability. GSEA identified enrichment of heparan sulfate biosynthesis and cAMP-responsive pathways in experimental models, findings that are presented as exploratory and that require prospective mechanistic validation. These findings provide a hypothesis-generating framework that may help inform clinical awareness and future mechanistic and prospective investigation.

HC-AEs demonstrate consistent and substantial occurrence during anthracycline therapy. Within the spectrum of anthracycline-related HC-AEs, embolism and thrombosis predominate as HLGT, primarily affecting respiratory, thoracic, mediastinal, neurological, vascular-lymphatic, and cardiac systems. The consistent identification of these adverse events in our pharmacovigilance analysis corroborates previous research regarding the thrombotic risks of anthracycline therapy. *In vitro* investigations have established the prothrombotic potential of anthracyclines, including doxorubicin and epirubicin, through their induction of procoagulant phenotypes in endothelial and mononuclear cells (Swystun et al., 2009). Clinical studies have documented elevated thrombosis rates in early-stage breast cancer patients receiving anthracycline therapy (Nolan et al., 2011). These observations are consistent with the positive signals for anthracycline-related HC-AEs identified in our analysis.

Drug-specific subgroup analyses showed that doxorubicin had the broadest coagulation toxicity signals among anthracyclines. This finding aligns with studies demonstrating a doxorubicin-induced procoagulant phenotype in endothelial and mononuclear cells. Mechanistic studies have shown that doxorubicin enhances platelet procoagulant activity via phosphatidylserine externalization and promotes thrombosis through multiple pathways, including reactive oxygen species (ROS), Ca2+-mediated microparticle production, adenosine triphosphate (ATP) depletion, and caspase-3 activation (Kim et al., 2011). Conversely, while daunorubicin displayed strong pharmacovigilance signals for cerebral venous and intracardiac thrombosis, these findings require cautious interpretation. The strong association may reflect, at least in part, the intrinsic hypercoagulable state associated with acute leukemia and the frequent use of concurrent pro-thrombotic therapies (e.g., L-asparaginase) in this patient population, making it difficult to isolate the specific contribution of daunorubicin itself (Tang et al., 2019; De Stefano et al., 2005; Hunault-Berger et al., 2008). Crucially, the universal requirement for central venous access in acute leukemia induction therapy introduces a significant mechanical confounder; catheter tips positioned within the right atrium are a well-documented cause of right atrial thrombosis, independent of drug toxicity (Korones et al., 1996; Picardi et al., 2023). Therefore, when interpreting the profound pharmacovigilance signal linking daunorubicin to intracardiac thrombosis, it is essential to explicitly decouple the potential drug-specific endothelial toxicity from CVC-induced mechanical thrombosis. As spontaneous adverse event reports cannot readily differentiate between these overlapping etiologies, this strong association must be interpreted with clinical caution. Nevertheless, anthracycline-induced cardiotoxicity provides a plausible indirect mechanism for intracardiac thrombosis. By impairing ventricular function, these drugs may promote intracardiac stasis, although direct causality remains unproven (Pai and Nahata, 2000). Additionally, epirubicin showed a distinct signal for thrombophlebitis, aligning with clinical observations of infusion-related chemical phlebitis (Williams et al., 2023). Collectively, these findings underscore drug-specific toxicity patterns, and may generate hypotheses to inform future prospective studies examining agent-specific and context-specific coagulation risk.

Our analysis showed that anthracycline-exposed patients experienced significantly earlier HC-AE onset (shorter TTO) than non-exposed patients, suggesting that anthracycline exposure may be associated with earlier onset of hypercoagulable events. Established research has described anthracycline-associated prothrombotic mechanisms, including endothelial cell injury, protein C pathway inhibition, tissue factor upregulation, and platelet activation, which collectively establish the molecular basis for early HC-AE onset (Grover et al., 2021; Swystun et al., 2009; Woodley-Cook et al., 2006; Lv et al., 2020; Chow et al., 2006). Although FAERS lacks comprehensive baseline clinical variables, our data show that age is significantly associated with differences in reported TTO: patients aged ≤39 years had substantially shorter TTO than older groups. This age-related temporal pattern aligns with prior reports emphasizing age as a critical modifier of thrombotic risk profiles in cancer populations. These results suggest that clinical surveillance strategies should be particularly alert to the rapid onset of HC-AEs during the early phases of treatment in younger patients.

Drug-specific differences in TTO likely reflect distinct procoagulant mechanisms and pharmacokinetics across anthracyclines. Daunorubicin has been shown to rapidly induce endothelial phosphatidylserine exposure and release of procoagulant microparticles, generating an early hypercoagulable milieu that may contribute to its shorter TTO (Fu et al., 2010). This biological pattern aligns with the clinical context of acute leukemia induction, where profound disease-related hypercoagulability and the near-universal use of intensive chemotherapy—with frequent central venous access—converge to produce a marked early spike in VTE incidence within the first days to weeks of treatment (Koschade et al., 2023). In contrast, epirubicin has different pharmacokinetics and greater tissue persistence, and has been shown to cause endothelial dysfunction and senescence, which may contribute to a delayed and more sustained vascular response consistent with its longer TTO (Shin et al., 2010; Eakin et al., 2020). This delayed pattern aligns with the cyclic nature of breast cancer adjuvant therapy, where thrombotic risk is cumulative and typically peaks within the first three treatment cycles (Kirwan et al., 2011). In addition, clinical factors—such as dose, infusion schedule, cisplatin-containing regimens, and catheter use—are known to modify thrombotic risk and timing and likely contribute to inter-drug heterogeneity in TTO (Oppelt et al., 2015).

When administered in combination regimens, the thrombotic risk profile of anthracyclines may be substantially altered. Clinical evidence confirms that doxorubicin-cyclophosphamide combination therapy significantly elevates thrombotic risk (Bjel et al., 2013). In multiple myeloma, doxorubicin-thalidomide regimens confer substantially higher DVT risk than thalidomide monotherapy (Zangari et al., 2002). While these agents may not be independently thrombogenic, their combination with anthracyclines substantially enhances thrombotic risk.

To explore the molecular context underlying these pharmacovigilance signals, we integrated pan-cancer transcriptomic analyses with *in vivo* validation. Across diverse human malignancies, we observed a preliminary negative correlation between HC-AE reporting odds and prostacyclin (PGI2) receptor signaling activity. Mechanistically, the prostacyclin receptor is a Gs protein–coupled receptor whose activation stimulates adenylyl cyclase, leading to increased intracellular cAMP levels and subsequent inhibition of platelet aggregation (Ruan and Dogné, 2006; Bill et al., 2014; Raslan et al., 2015; Stitham et al., 2007). Notably, the reduced prostacyclin receptor signaling activity observed in TCGA cancer cohorts may reflect a biological context that is consistent with the GSEA-identified suppression of cAMP-related signaling pathways in our murine model. Taken together, these exploratory observations raise the hypothesis that perturbation of the prostacyclin–cAMP signaling axis may be associated with anthracycline-related hypercoagulability, a possibility that warrants prospective mechanistic investigation. From a translational perspective, the observed association with cAMP-related signaling raises the hypothesis that pharmacologic modulation of intracellular cAMP—such as through phosphodiesterase (PDE) inhibition—represents a strictly theoretical and highly speculative avenue at this stage, the translational relevance of which remains entirely unestablished and would require extensive prospective mechanistic and clinical validation before any therapeutic application could be considered (Gresele et al., 2011).

It is important to acknowledge, however, that the transcriptomic alterations observed in our model reflect cumulative drug exposure following 4 weeks of continuous dosing, rather than the response to any single plasma peak. While systemic clearance of anthracyclines is relatively rapid, tissue persistence beyond plasma half-life has been documented (Shin et al., 2010). Furthermore, transient doxorubicin exposure has been shown to trigger endothelial senescence that progresses and persists after drug withdrawal (Graziani et al., 2022), a phenotype recently proven to directly mediate sustained doxorubicin-induced vascular endothelial dysfunction (Venkatasubramanian et al., 2025). A more direct mechanistic basis for this cAMP suppression may reside in the prostacyclin biosynthetic pathway: high intracellular ROS concentrations generated by doxorubicin metabolism have been shown to inhibit the cyclooxygenase step of PGI2 synthesis in endothelial cells (Luu et al., 2018), directly reducing IP receptor activation and downstream cAMP levels—a mechanism that may be re-triggered with each successive chemotherapy cycle, providing a molecular basis for cumulative cAMP pathway suppression consistent with both our GSEA findings and the pan-cancer prostacyclin receptor correlation. Whether such persistent endothelial reprogramming contributes to sustained cAMP signaling suppression and the delayed thrombotic events observed in our TTO analysis remains an important question for future dedicated mechanistic investigation.

In parallel, we identified downregulation of heparan sulfate biosynthesis pathways, which have been implicated in maintaining endothelial anticoagulant properties and may further contribute to a prothrombotic milieu (Sobczak et al., 2018; Nadir, 2021). This observation warrants critical interpretation within the context of our tumor-bearing model. Heparan sulfate (HS) constitutes the predominant glycosaminoglycan component of the endothelial glycocalyx and is essential for vascular thromboresistance through its roles in antithrombin III activation and sequestration of procoagulant adhesion molecules from the endothelial surface (Reitsma et al., 2007). Two non-mutually exclusive mechanisms may account for this transcriptomic signal. First, a drug-direct effect is well-supported: doxorubicin induces endothelial oxidative stress via excessive mitochondrial reactive oxygen species generation, causing endothelial dysfunction and glycocalyx injury (Clayton et al., 2020). Such ROS-mediated damage can impair HS proteoglycan biosynthesis and promote glycocalyx shedding, directly compromising the endothelial anticoagulant surface. Second, the melanoma microenvironment itself independently drives HS degradation: B16-F10 tumor cells constitutively release tissue factor (TF)-bearing microparticles that accelerate thrombosis (de Oliveira et al., 2012; Muhsin-Sharafaldine et al., 2016), while tumor-derived heparanase enzymatically cleaves HS chains, further disrupting glycocalyx integrity (Nadir, 2021). Therefore, the observed suppression of HS pathways may reflect a synergistic dynamic in which anthracycline-induced oxidative endothelial injury acts as a second hit upon a glycocalyx already compromised by tumor-mediated heparanase activity. However, the current experimental design—lacking a tumor-free drug-treated control arm and direct glycocalyx integrity measurements—does not permit formal separation of these two contributions. Future studies incorporating such designs will be necessary to resolve this mechanistic question.

Beyond heparan sulfate remodeling, the exacerbated coagulation phenotype in doxorubicin-treated tumor-bearing animals likely reflects a dynamic interplay between tumor-derived and drug-induced procoagulant stimuli. Melanoma cells constitutively express TF and release TF-bearing microparticles that establish a Trousseau-like hypercoagulable baseline (de Oliveira et al., 2012; Muhsin-Sharafaldine et al., 2016). Doxorubicin independently amplifies this baseline by inducing TF expression and phosphatidylserine externalization on host endothelial cells and monocytes (Swystun et al., 2009). Notably, Algarni et al. demonstrated in microfluidic *ex vivo* models that these two stimuli are not merely additive: doxorubicin significantly enhances the procoagulant activity of endothelial cells previously conditioned by tumor microparticles (Algarni et al., 2020; Antoniak et al., 2021); this convergent procoagulant dynamic is further supported by Zhang et al., who showed that doxorubicin induces the release of procoagulant phosphatidylserine-exposing cancer cell-derived microparticles that directly convert endothelial cells to a procoagulant phenotype—an effect confirmed in clinical samples from patients receiving doxorubicin-based chemotherapy (Zhang et al., 2021). The statistically significant incremental elevations in FIB, PCT, and PLT in our doxorubicin-treated cohort above the PBS-treated tumor-bearing baseline are directionally consistent with this synergistic model, though formal in-model quantification requires future factorial designs incorporating serial TF profiling.

Complementing this primary axis, GSEA of an external rat transcriptomic dataset (GSE37260) revealed enrichment of two additional coagulation-relevant pathways: transcriptional regulation by RUNX1 and IL-1 family cytokine signaling. RUNX1 is a central regulator of hematopoiesis and megakaryocyte differentiation and has been implicated in platelet biology and coagulation-related gene expression, including *F13A1*-mediated clot stabilization and the inhibition of fibrinolysis (Yuan et al., 2023; Del Carpio-Cano et al., 2025; Xiao et al., 2024). These observations suggest that altered RUNX1-associated transcriptional programs may contribute to procoagulant phenotypes in the context of anthracycline exposure. Concurrently, IL-1β signaling is well recognized to induce a prothrombotic endothelial phenotype and to promote inflammation-associated thrombus formation (Puhlmann et al., 2005). Collectively, these data suggest that coordinated transcriptional and inflammatory mechanisms may further amplify hypercoagulable states associated with anthracyclines.

Consistent with these molecular associations, our retrospective clinical analysis identified trends suggestive of hypercoagulability following doxorubicin administration, reflected by alterations in AT-III and PTA levels. In parallel murine experiments, doxorubicin-treated animals exhibited comparable procoagulant tendencies, including significantly elevated FIB levels. Hematological analyses also revealed increased PCT and PLT values relative to controls. These findings are in line with prior preclinical studies reporting that doxorubicin can promote platelet activation, enhance platelet–endothelial interactions (Lv et al., 2020; Ben Aharon et al., 2013), and increase thrombin–antithrombin complex formation and cell-free deoxyribonucleic acid (DNA)-mediated thrombin generation in a dose-dependent manner (Swystun et al., 2011). Collectively, existing evidence supports the notion that anthracyclines may influence coagulation homeostasis through multiple mechanisms, including phosphatidylserine externalization, extracellular vesicle release, modulation of the protein C pathway, and vascular injury (Wang et al., 2023; Swystun et al., 2009; Woodley-Cook et al., 2006; Ben Aharon et al., 2013; Bar-Joseph et al., 2015; Yarana et al., 2018; Aubertin et al., 2016). While malignancy-related factors undoubtedly contribute to coagulation imbalance, the consistent association between doxorubicin exposure and procoagulant alterations observed across analytical layers warrants further prospective investigation.

Several limitations should be considered in the interpretation of this study. The FAERS database, as a spontaneous reporting system, presents inherent selection biases, including geographical reporting disparities, temporal variations in drug availability, and incomplete reporting, limiting causal inference and incidence estimation of anthracycline-related HC-AEs (Zhou et al., 2023). VigiBase’s absence of data regarding dosing regimens, clinical indications, mortality timing, and other crucial parameters precludes comprehensive competing risk analyses (Storck et al., 2024). The heterogeneity of antineoplastic agents complicates control group establishment, potentially introducing indication bias. Disproportionality analysis results are constrained by reporting patterns, marketing influences, and drug utilization scope, reflecting only reported statistics rather than actual incidence or risk (Storck et al., 2024; Qu et al., 2024). Additional limitations include insufficient mechanistic evidence and the absence of key clinical parameters (drug dosing, treatment duration, administration timing, and tapering protocols), necessitating validation through large-scale prospective studies. Furthermore, the retrospective clinical cohort has several important limitations. The single-center design and retrospective nature preclude causal interpretation. Crucially, several key confounding variables were not systematically collected or adjusted for, including cancer type and stage, specific anthracycline regimen and cumulative dose, concurrent chemotherapy agents (such as cyclophosphamide or thalidomide, which independently elevate thrombotic risk), central venous catheter status, baseline thrombotic risk (e.g., Khorana score), and relevant comorbidities. These factors are known independent determinants of coagulation dynamics and represent sources of residual confounding that cannot be excluded in the present analysis. The observed changes in AT-III and PTA levels should therefore be interpreted as exploratory trends associated with anthracycline exposure in this clinical context, rather than as evidence of drug-specific effects. Therefore, these findings should be regarded as exploratory observations that complement, rather than independently validate, the pharmacovigilance and experimental results. Regarding our *in vivo* experiments, the findings are constrained by a small sample size, single-sex design, and reliance on a specific murine melanoma model, which may not fully represent the pan-cancer hypercoagulable complexity in humans. Moreover, the absence of a tumor-free doxorubicin-treated control arm precludes formal separation of drug-direct from tumor-mediated contributions to the observed suppression of heparan sulfate biosynthesis pathways, an interpretive limitation that future factorial experimental designs should address. Similarly, the lack of dynamic tissue factor (TF) and microparticle profiling prevents the formal *in vivo* decoupling of drug-induced and tumor-derived prothrombotic mechanisms. Finally, the pan-cancer transcriptomic correlation remains strictly exploratory; its reliance on a limited number of cancer types makes the statistical findings vulnerable to outliers, precluding definitive biological conclusions. These factors underscore the necessity for future multicenter prospective validation.

## Conclusion

Systematic analysis of the FAERS and VigiBase databases revealed elevated HC-AE reporting frequencies in anthracycline-treated oncology patients. These findings are hypothesis-generating and do not establish incidence rates, absolute risk, or causality. Large-scale prospective studies are warranted to further characterize the true incidence, underlying mechanisms, and associated risk factors.

## Data Availability

The original contributions presented in the study are included in the article/Supplementary Material, further inquiries can be directed to the corresponding author.

## Glossary

AEs: adverse events
APTT: activated partial thromboplastin time
AT: arterial thrombosis
AT-III: antithrombin III
ATP: adenosine triphosphate
cAEs: coagulation-related AEs
cAMP: cyclic adenosine monophosphate
CI: confidence interval
CVT: cerebral venous thrombosis
DMEM: Dulbecco’s Modified Eagle Medium
DNA: deoxyribonucleic acid
DNR: daunorubicin
DOX: doxorubicin
DVT: deep vein thrombosis
EMA: European Medicines Agency
EPI: epirubicin
FAERS: the FDA Adverse Event Reporting System
FDA: Food and Drug Administration
FIB: fibrinogen
FPKM: Fragments Per Kilobase of transcript per Million mapped reads
GEO: Gene Expression Omnibus
GO: Gene Ontology
GSEA: Gene Set Enrichment Analysis
GSVA: Gene Set Variation Analysis
HC-AEs: hypercoagulable adverse events
HLGT: higher-level group terms
HLT: high-level terms
HS: heparan sulfate
IL-1: interleukin-1
IQR: interquartile ranges
KEGG: Kyoto Encyclopedia of Genes and Genomes
**log**_**2**_ **FC**: log2 fold change
MedDRA: the Medical Dictionary for Regulatory Activities
MM: multiple myeloma
MSigDB: Molecular Signatures Database
NES: normalized enrichment scores
OV: ovarian cancer
PAI-1: plasminogen activator inhibitor-1
PCT: plateletcrit
PBS: phosphate-buffered saline
PDW: platelet distribution width
PE: pulmonary embolism
PLT: platelet count
PRR: proportional reporting ratio
PS: primary suspect
PT: preferred terms
PT: prothrombin time
PTA: prothrombin activity
RNA: ribonucleic acid
ROR: reporting odds ratio
ROS: reactive oxygen species
SOCs: system organ classes
SPF: specific pathogen-free
ssGSEA: Single-Sample Gene Set Enrichment Analysis
TCGA: The Cancer Genome Atlas
TED: thromboembolic disease
TF: tissue factor
TPM: transcripts per million
TTO: time-to-onset
**UCSC**University of California: University of CaliforniaSanta Cruz
VigiBase: the world health organization’s global individual case safety report database.

## References

[B1] Abdol Razak N. B. Jones G. Bhandari M. Berndt M. C. Metharom P. (2018). Cancer-associated thrombosis: an overview of mechanisms, risk factors, and treatment. Cancers 10 (10), 380. 10.3390/cancers10100380 30314362 PMC6209883

[B2] Algarni A. Greenman J. Madden L. A. (2020). Doxorubicin enhances procoagulant activity of endothelial cells after exposure to tumour microparticles on microfluidic devices. Hemato 1 (1), 23–34. 10.3390/bloods1010006

[B3] Antoniak S. Phungphong S. Cheng Z. Jensen B. C. (2021). Novel mechanisms of anthracycline-induced cardiovascular toxicity: a focus on thrombosis, cardiac atrophy, and programmed cell death. Front. Cardiovascular Medicine 8, 817977. 10.3389/fcvm.2021.817977 35111832 PMC8801506

[B4] Aubertin K. Silva A. K. Luciani N. Espinosa A. Djemat A. Charue D. (2016). Massive release of extracellular vesicles from cancer cells after photodynamic treatment or chemotherapy. Sci. Reports 6, 35376. 10.1038/srep35376 27752092 PMC5067517

[B5] Bar-Joseph H. Stemmer S. M. Tsarfaty I. Shalgi R. Ben-Aharon I. (2015). Chemotherapy-induced vascular toxicity--real-time *in vivo* imaging of vessel impairment. J. Vis. Exp. (95), e51650. 10.3791/51650 25590564 PMC4354501

[B6] Barbie D. A. Tamayo P. Boehm J. S. Kim S. Y. Moody S. E. Dunn I. F. (2009). Systematic RNA interference reveals that oncogenic KRAS-Driven cancers require TBK1. Nature 462 (7269), 108–112. 10.1038/nature08460 19847166 PMC2783335

[B7] Ben Aharon I. Bar Joseph H. Tzabari M. Shenkman B. Farzam N. Levi M. (2013). Doxorubicin-induced vascular toxicity--targeting potential pathways May reduce procoagulant activity. PloS One 8 (9), e75157. 10.1371/journal.pone.0075157 24073244 PMC3779248

[B8] Bill A. Rosethorne E. M. Kent T. C. Fawcett L. Burchell L. van Diepen M. T. (2014). High throughput mutagenesis for identification of residues regulating human prostacyclin (hIP) receptor expression and function. PloS One 9 (6), e97973. 10.1371/journal.pone.0097973 24886841 PMC4041722

[B9] Bjelogrlic S. K. Lukic S. T. Djuricic S. M. (2013). Activity of dexrazoxane and amifostine against late cardiotoxicity induced by the combination of doxorubicin and cyclophosphamide in vivo. Basic and Clinical Pharmacology and Toxicology 113 (4), 228–238. 10.1111/bcpt.12086 23692343

[B10] Caster O. Aoki Y. Gattepaille L. M. Grundmark B. (2020). Disproportionality analysis for pharmacovigilance signal detection in small databases or subsets: recommendations for limiting false-positive associations. Drug Safety 43 (5), 479–487. 10.1007/s40264-020-00911-w 32008183 PMC7165139

[B11] Chow A. Y. Chin C. Dahl G. Rosenthal D. N. (2006). Anthracyclines cause endothelial injury in pediatric cancer patients: a pilot study. J. Clinical Oncology Official Journal Am. Soc. Clin. Oncol. 24 (6), 925–928. 10.1200/JCO.2005.03.5956 16484703

[B12] Clayton Z. S. Brunt V. E. Hutton D. A. VanDongen N. S. D’Alessandro A. Reisz J. A. (2020). Doxorubicin-induced oxidative stress and endothelial dysfunction in conduit arteries is prevented by mitochondrial-specific antioxidant treatment. JACC CardioOncology 2 (3), 475–488. 10.1016/j.jaccao.2020.06.010 33073250 PMC7561020

[B13] de Oliveira A. D. S. Lima L. G. Mariano-Oliveira A. Machado D. E. Nasciutti L. E. Andersen J. F. (2012). Inhibition of tissue factor by ixolaris reduces primary tumor growth and experimental metastasis in a murine model of melanoma. Thrombosis Research 130 (3), e163–e170. 10.1016/j.thromres.2012.05.021 22683021 PMC3424357

[B14] De Stefano V. Sorà F. Rossi E. Chiusolo P. Laurenti L. Fianchi L. (2005). The risk of thrombosis in patients with acute leukemia: occurrence of thrombosis at diagnosis and during treatment. J. Thrombosis Haemostasis JTH 3 (9), 1985–1992. 10.1111/j.1538-7836.2005.01467.x 16102104

[B15] Del Carpio-Cano F. Songdej N. Guan L. Mao G. Goldfinger L. E. Wurtzel J. G. T. (2025). Transcription factor RUNX1 regulates coagulation factor XIII-A (F13A1): decreased platelet-megakaryocyte F13A1 expression and clot contraction in RUNX1 haplodeficiency. Res. Practice Thrombosis Haemostasis 9 (1), 102680. 10.1016/j.rpth.2025.102680 PMC11849627 39995753

[B16] Eakin A. J. Mc Erlain T. Burke A. Eaton A. Tipping N. Allocca G. (2020). Circulating levels of epirubicin cause endothelial senescence while compromising metabolic activity and vascular function. Front. Cell Developmental Biology 8, 799. 10.3389/fcell.2020.00799 32974345 PMC7466755

[B17] Fu Y. Zhou J. Li H. Cao F. Su Y. Fan S. (2010). Daunorubicin induces procoagulant activity of cultured endothelial cells through phosphatidylserine exposure and microparticles release. Thrombosis Haemostasis 104 (6), 1235–1241. 10.1160/TH10-02-0102 20886178

[B18] Gastaldon C. Arzenton E. Raschi E. Spigset O. Papola D. Ostuzzi G. (2023). Neonatal withdrawal syndrome following in utero exposure to antidepressants: a disproportionality analysis of VigiBase, the WHO spontaneous reporting database. Psychol. Medicine 53 (12), 5645–5653. 10.1017/s0033291722002859 36128628 PMC10482711

[B19] Goldman M. J. Craft B. Hastie M. Repečka K. McDade F. Kamath A. (2020). Visualizing and interpreting cancer genomics data via the xena platform. Nat. Biotechnology 38 (6), 675–678. 10.1038/s41587-020-0546-8 32444850 PMC7386072

[B20] Goodnough L. T. Saito H. Manni A. Jones P. K. Pearson O. H. (1984). Increased incidence of thromboembolism in stage IV breast cancer patients treated with a five-drug chemotherapy regimen. A study of 159 patients. Cancer 54 (7), 1264–1268. 10.1002/1097-0142(19841001)54:7<1264::aid-cncr2820540706>3.0.co;2-r 6547874

[B21] Graziani S. Scorrano L. Pontarin G. (2022). Transient exposure of endothelial cells to doxorubicin leads to long-lasting vascular endothelial growth factor receptor 2 downregulation. Cells 11 (2), 210. 10.3390/cells11020210 35053325 PMC8773916

[B22] Gresele P. Momi S. Falcinelli E. (2011). Anti-platelet therapy: phosphodiesterase inhibitors. Br. Journal Clinical Pharmacology 72 (4), 634–646. 10.1111/j.1365-2125.2011.04034.x 21649691 PMC3195739

[B23] Grover S. P. Hisada Y. M. Kasthuri R. S. Reeves B. N. Mackman N. (2021). Cancer therapy-associated thrombosis. Arteriosclerosis, Thrombosis, Vascular Biology 41 (4), 1291–1305. 10.1161/atvbaha.120.314378 33567864 PMC7990713

[B24] Hänzelmann S. Castelo R. Guinney J. (2013). GSVA: gene set variation analysis for microarray and RNA-Seq data. BMC Bioinformatics 14, 7. 10.1186/1471-2105-14-7 23323831 PMC3618321

[B25] Hisada Y. Mackman N. (2017). Cancer-associated pathways and biomarkers of venous thrombosis. Blood 130 (13), 1499–1506. 10.1182/blood-2017-03-743211 28807983 PMC5620413

[B26] Hunault-Berger M. Chevallier P. Delain M. Bulabois C. E. Bologna S. Bernard M. (2008). Changes in antithrombin and fibrinogen levels during induction chemotherapy with L-asparaginase in adult patients with acute lymphoblastic leukemia or lymphoblastic lymphoma. Use of supportive coagulation therapy and clinical outcome: the CAPELAL study. Haematologica 93 (10), 1488–1494. 10.3324/haematol.12948 18728028

[B27] Jing Y. Liu J. Ye Y. Pan L. Deng H. Wang Y. (2020). Multi-omics prediction of immune-related adverse events during checkpoint immunotherapy. Nat. Communications 11 (1), 4946. 10.1038/s41467-020-18742-9 33009409 PMC7532211

[B28] Key N. S. Khorana A. A. Kuderer N. M. Bohlke K. Lee A. Y. Y. Arcelus J. I. (2020). Venous thromboembolism prophylaxis and treatment in patients with cancer: ASCO clinical practice guideline update. J. Clinical Oncology Official Journal Am. Soc. Clin. Oncol. 38 (5), 496–520. 10.1200/JCO.19.01461 31381464

[B29] Khaleel M. A. Khan A. H. Ghadzi S. M. S. Adnan A. S. Abdallah Q. M. (2022). A standardized dataset of a spontaneous adverse event reporting system. Healthc. Basel, Switz. 10 (3), 420. 10.3390/healthcare10030420 35326898 PMC8954498

[B30] Kim S. H. Lim K. M. Noh J. Y. Kim K. Kang S. Chang Y. K. (2011). Doxorubicin-induced platelet procoagulant activities: an important clue for chemotherapy-associated thrombosis. Toxicol. Sciences An Official Journal Soc. Toxicol. 124 (1), 215–224. 10.1093/toxsci/kfr222 21865289

[B31] Kirwan C. C. McDowell G. McCollum C. N. Byrne G. J. (2011). Incidence of venous thromboembolism during chemotherapy for breast cancer: impact on cancer outcome. Anticancer Research 31 (6), 2383–2388. Available online at: https://pubmed.ncbi.nlm.nih.gov/21737669/. 21737669

[B32] Korones D. N. Buzzard C. J. Asselin B. L. Harris J. P. (1996). Right atrial thrombi in children with cancer and indwelling catheters. J. Pediatrics 128 (6), 841–846. 10.1016/s0022-3476(96)70338-4 8648545

[B33] Koschade S. E. Stratmann J. A. Steffen B. Shaid S. Finkelmeier F. Serve H. (2023). Early-onset venous thromboembolisms in newly diagnosed non-promyelocytic acute myeloid leukemia patients undergoing intensive induction chemotherapy. Eur. Journal Haematology 110 (4), 426–434. 10.1111/ejh.13920 36573351

[B34] Li H. Sun X. Sun D. Zhao J. Xu Z. Zhao P. (2021). Thromboembolic events associated with immune checkpoint inhibitors: a real-world study of data from the food and drug administration adverse event reporting system (FAERS) database. Int. Immunopharmacology 98, 107818. 10.1016/j.intimp.2021.107818 34130149

[B35] Lindquist M. (2008). VigiBase, the WHO global ICSR database system: basic facts. Drug Inf. J. 42 (5), 409–419. 10.1177/009286150804200501

[B36] Luu A. Z. Chowdhury B. Al-Omran M. Teoh H. Hess D. A. Verma S. (2018). Role of endothelium in doxorubicin-induced cardiomyopathy. JACC Basic Translational Science 3 (6), 861–870. 10.1016/j.jacbts.2018.06.005 30623145 PMC6314956

[B37] Lv H. Tan R. Liao J. Hao Z. Yang X. Liu Y. (2020). Doxorubicin contributes to thrombus formation and vascular injury by interfering with platelet function. Am. Journal Physiology Heart Circulatory Physiology 319 (1), H133. 10.1152/ajpheart.00456.2019 32469636

[B38] Lyon A. R. López-Fernández T. Couch L. S. Asteggiano R. Aznar M. C. Bergler-Klein J. (2022). 2022 ESC guidelines on cardio-oncology developed in collaboration with the European Hematology Association (EHA), the European Society for Therapeutic Radiology and Oncology (ESTRO) and the International Cardio-Oncology Society (IC-OS). Eur. Heart Journal 43 (41), 4229–4361. 10.1093/eurheartj/ehac244 36017568

[B39] Mattioli R. Ilari A. Colotti B. Mosca L. Fazi F. Colotti G. (2023). Doxorubicin and other anthracyclines in cancers: activity, chemoresistance and its overcoming. Mol. Aspects Medicine 93, 101205. 10.1016/j.mam.2023.101205 37515939

[B40] Muhsin-Sharafaldine M. R. Saunderson S. C. Dunn A. C. Faed J. M. Kleffmann T. McLellan A. D. (2016). Procoagulant and immunogenic properties of melanoma exosomes, microvesicles and apoptotic vesicles. Oncotarget 7 (35), 56279–56294. 10.18632/oncotarget.10783 27462921 PMC5302914

[B41] Nadir Y. (2021). Effect of heparanase and heparan sulfate chains in hemostasis. Seminars Thrombosis Hemostasis. 47 (3), 254–260. 10.1055/s-0041-1725065 33794550

[B42] Nolan L. Darby A. Boleti K. Simmonds P. (2011). The incidence of symptomatic thromboembolism in patients receiving adjuvant anthracycline-based chemotherapy for early stage breast cancer. Breastedinbg. Scotl. 20 (2), 151–154. 10.1016/j.breast.2010.09.001 20970333

[B43] Oppelt P. Betbadal A. Nayak L. (2015). Approach to chemotherapy-associated thrombosis. Vasc. Medicine Lond. Engl. 20 (2), 153–161. 10.1177/1358863X14568705 25832603 PMC4655120

[B44] Pai V. B. Nahata M. C. (2000). Cardiotoxicity of chemotherapeutic agents: incidence, treatment and prevention. Drug Safety 22 (4), 263–302. 10.2165/00002018-200022040-00002 10789823

[B45] Picardi M. Giordano C. Della Pepa R. Pugliese N. Esposito M. Abagnale D. P. (2023). Intravascular complications of central venous catheterization by insertion site in acute leukemia during remission induction chemotherapy phase: lower risk with peripherally inserted catheters in a single-center retrospective study. Cancers 15 (7), 2147. 10.3390/cancers15072147 37046808 PMC10093126

[B46] Puhlmann M. Weinreich D. M. Farma J. M. Carroll N. M. Turner E. M. Alexander H. R. Jr (2005). Interleukin-1beta induced vascular permeability is dependent on induction of endothelial tissue factor (TF) activity. J. Translational Medicine 3, 37. 10.1186/1479-5876-3-37 16197553 PMC1276820

[B47] Qu H. Li W. Wu Z. Wang Y. Feng T. Li N. (2024). Differences in hypersensitivity reactions and gadolinium deposition disease/symptoms associated with gadolinium exposure to gadolinium-based contrast agents: new insights based on global databases VigiBase, FAERS, and IQVIA-MIDAS. BMC Medicine 22 (1), 329. 10.1186/s12916-024-03537-2 39135199 PMC11321222

[B48] Raslan Z. Aburima A. Naseem K. M. (2015). The spatiotemporal regulation of cAMP signaling in blood platelets-old friends and new players. Front. Pharmacology 6, 266. 10.3389/fphar.2015.00266 26617518 PMC4639615

[B49] Reitsma S. Slaaf D. W. Vink H. van Zandvoort M. A. oude Egbrink M. G. (2007). The endothelial glycocalyx: composition, functions, and visualization. Pflugers Archiv Eur. Journal Physiology 454 (3), 345–359. 10.1007/s00424-007-0212-8 17256154 PMC1915585

[B50] Ruan K. H. Dogné J. M. (2006). Implications of the molecular basis of prostacyclin biosynthesis and signaling in pharmaceutical designs. Curr. Pharmaceutical Design 12 (8), 925–941. 10.2174/138161206776055994 16533160

[B51] Sakaeda T. Kadoyama K. Okuno Y. (2011). Statin-associated muscular and renal adverse events: data mining of the public version of the FDA adverse event reporting system. PloS One 6 (12), e28124. 10.1371/journal.pone.0028124 22205938 PMC3243683

[B52] Semchuk W. M. Sperlich C. (2012). Prevention and treatment of venous thromboembolism in patients with cancer. Can. Pharm. J. CPJ = Revue des Pharm. du Can. RPC 145 (1), 24. 10.3821/1913-701X-145.1.24 23509484 PMC3567538

[B53] Shin M. Matsunaga H. Fujiwara K. (2010). Differences in accumulation of anthracyclines daunorubicin, doxorubicin and epirubicin in rat tissues revealed by immunocytochemistry. Histochem. Cell Biology 133 (6), 677–682. 10.1007/s00418-010-0700-3 20424853

[B54] Sobczak A. I. S. Pitt S. J. Stewart A. J. (2018). Glycosaminoglycan neutralization in coagulation control. Arteriosclerosis, Thrombosis, Vascular Biology 38 (6), 1258–1270. 10.1161/ATVBAHA.118.311102 29674476 PMC5965931

[B55] Sørensen H. T. Pedersen L. van Es N. Büller H. R. Horváth-Puhó E. (2023). Impact of venous thromboembolism on the mortality in patients with cancer: a population-based cohort study. Lancet Regional Health Eur. 34, 100739. 10.1016/j.lanepe.2023.100739 37809052 PMC10558815

[B56] Stitham J. Arehart E. J. Gleim S. R. Douville K. L. Hwa J. (2007). Human prostacyclin receptor structure and function from naturally-occurring and synthetic mutations. Prostagl. and Other Lipid Mediators 82 (1-4), 95–108. 10.1016/j.prostaglandins.2006.05.010 17164137

[B57] Storck W. de Laportalière T. T. Yrondi A. Javelot H. Berna F. Montastruc F. (2024). Withdrawal syndrome after antipsychotics discontinuation: an analysis of the WHO database of spontaneous reports (vigibase) between 2000 and 2022. Psychopharmacology 241 (6), 1205–1212. 10.1007/s00213-024-06554-4 38376511

[B58] Subramanian A. Tamayo P. Mootha V. K. Mukherjee S. Ebert B. L. Gillette M. A. (2005). Gene set enrichment analysis: a knowledge-based approach for interpreting genome-wide expression profiles. Proc. Natl. Acad. Sci. U. S. A. 102 (43), 15545–15550. 10.1073/pnas.0506580102 16199517 PMC1239896

[B59] Swystun L. L. Shin L. Y. Beaudin S. Liaw P. C. (2009). Chemotherapeutic agents doxorubicin and epirubicin induce a procoagulant phenotype on endothelial cells and blood monocytes. J. Thrombosis Haemostasis JTH 7 (4), 619–626. 10.1111/j.1538-7836.2009.03300.x 19187077

[B60] Swystun L. L. Mukherjee S. Liaw P. C. (2011). Breast cancer chemotherapy induces the release of cell-free DNA, a novel procoagulant stimulus. J. Thrombosis Haemostasis JTH 9 (11), 2313–2321. 10.1111/j.1538-7836.2011.04465.x 21838758

[B61] Tang A. S. O. Yeo S. T. Law W. C. Chew L. P. (2019). Cerebral venous thrombosis as an initial manifestation of acute myeloid leukemia. Oxf. Medical Case Reports 2019 (1), omy118. 10.1093/omcr/omy118 30697435 PMC6345084

[B62] Thomas R. H. (2001). Hypercoagulability syndromes. Archives Internal Medicine 161 (20), 2433–2439. 10.1001/archinte.161.20.2433 11700155

[B63] Todorova V. K. Beggs M. L. Delongchamp R. R. Dhakal I. Makhoul I. Wei J. Y. (2012). Transcriptome profiling of peripheral blood cells identifies potential biomarkers for doxorubicin cardiotoxicity in a rat model. PloS One 7 (11), e48398. 10.1371/journal.pone.0048398 23209553 PMC3507887

[B64] Tuzovic M. Wu P. T. Kianmahd S. Nguyen K. L. (2018). Natural history of myocardial deformation in children, adolescents, and young adults exposed to anthracyclines: systematic review and meta-analysis. Echocardiogr. Mt. Kisco, NY 35 (7), 922–934. 10.1111/echo.13871 29603386 PMC6544758

[B65] Venkatasubramanian R. Mahoney S. A. Hutton D. A. VanDongen N. S. Brunt V. E. Greenberg N. T. (2025). Cellular senescence mediates doxorubicin chemotherapy-induced vascular endothelial dysfunction: translational evidence of prevention with senolytic treatment. Am. J. Physiology-Heart Circulatory Physiology 329 (6), H1672–H1683. 10.1152/ajpheart.00712.2025 41143747 PMC12704655

[B66] Wagner G. P. Kin K. Lynch V. J. (2012). Measurement of mRNA abundance using RNA-Seq data: RPKM measure is inconsistent among samples. Theory Biosciences = Theor. Den Biowiss. 131 (4), 281–285. 10.1007/s12064-012-0162-3 22872506

[B67] Wang T. H. Ma Y. Gao S. Zhang W. W. Han D. Cao F. (2023). Recent advances in the mechanisms of cell death and dysfunction in doxorubicin cardiotoxicity. Rev. Cardiovascular Medicine 24 (11), 336. 10.31083/j.rcm2411336 39076437 PMC11272847

[B68] Williams N. Williams E. M. Roberts R. (2023). Chemotherapy induced phlebitis experienced by women with breast cancer following administration of epirubicin using a volumetric infusion pump: an observational study. Eur. Journal Oncology Nursing The Official Journal Eur. Oncol. Nurs. Soc. 64, 102322. 10.1016/j.ejon.2023.102322 37141665

[B69] Woodley-Cook J. Shin L. Y. Swystun L. Caruso S. Beaudin S. Liaw P. C. (2006). Effects of the chemotherapeutic agent doxorubicin on the protein C anticoagulant pathway. Mol. Cancer Therapeutics 5 (12), 3303–3311. 10.1158/1535-7163.MCT-06-0154 17172434

[B70] Xiao C. Liu J. Cheng Y. Wu Y. Li Q. Chen X. (2024). RUNX1 targeting AKT3 promotes alveolar hypercoagulation and fibrinolytic inhibition in LPS induced ARDS. Respir. Research 25 (1), 54. 10.1186/s12931-024-02689-2 38267920 PMC10809548

[B71] Yan Y. D. Zhao Y. Zhang C. Fu J. Su Y. J. Cui X. L. (2022). Toxicity spectrum of immunotherapy in advanced lung cancer: a safety analysis from clinical trials and a pharmacovigilance system. EClinicalMedicine 50, 101535. 10.1016/j.eclinm.2022.101535 35812997 PMC9256649

[B72] Yan X. Li Z. Jiang A. Chen J. Huang X. Hajdu A. (2025). Immunotherapy-induced cholestasis in cancer: insights from the two real-world pharmacovigilance databases of FAERS and VigiBase. Int. Journal Surgery Lond. Engl. 111 (8), 5105–5121. 10.1097/js9.0000000000002607 40479008

[B73] Yarana C. Carroll D. Chen J. Chaiswing L. Zhao Y. Noel T. (2018). Extracellular vesicles released by cardiomyocytes in a doxorubicin-induced cardiac injury mouse model contain protein biomarkers of early cardiac injury. Clin. Cancer Research An Official Journal Am. Assoc. Cancer Res. 24 (7), 1644–1653. 10.1158/1078-0432.CCR-17-2046 29070527 PMC6193451

[B74] Yuan H. Liu Y. Zhang J. Dong J. F. Zhao Z. (2023). Transcription factors in megakaryocytes and platelets. Front. Immunology 14, 1140501. 10.3389/fimmu.2023.1140501 36969155 PMC10034027

[B75] Zangari M. Siegel E. Barlogie B. Anaissie E. Saghafifar F. Fassas A. (2002). Thrombogenic activity of doxorubicin in myeloma patients receiving thalidomide: implications for therapy. Blood 100 (4), 1168–1171. 10.1182/blood-2002-01-0335 12149193

[B76] Zhang C. Yang Z. Zhou P. Yu M. Li B. Liu Y. (2021). Phosphatidylserine-exposing tumor-derived microparticles exacerbate coagulation and cancer cell transendothelial migration in triple-negative breast cancer. Theranostics 11 (13), 6445–6460. 10.7150/thno.53637 33995667 PMC8120203

[B77] Zhang J. Li H. Tao W. Zhou JJMR (2025). Gseavis: An R Package for Enhanced Visualization of Gene Set Enrichment Analysis in Biomedicine.

[B78] Zhou C. Peng S. Lin A. Jiang A. Peng Y. Gu T. (2023). Psychiatric disorders associated with immune checkpoint inhibitors: a pharmacovigilance analysis of the FDA Adverse Event Reporting System (FAERS) database. EClinicalMedicine 59, 101967. 10.1016/j.eclinm.2023.101967 37131541 PMC10149185

