# Supplementary material for: From signal to significance: characterizing anthracycline-related hypercoagulable adverse events via pharmacovigilance, animal models, and clinical observation
Source: Front Pharmacol. 2026 Jul 31;17:1856410. doi: 10.3389/fphar.2026.1856410 (PMC13473302; doi:10.3389/fphar.2026.1856410)
Supplement: Supplementary file 1 [file DataSheet1.docx]

**From Signal to Significance: Characterizing Anthracycline-Related Hypercoagulable Adverse Events via Pharmacovigilance, Animal Models, and Clinical Validation**

| **Principal Investigator** | Jian Zhang; Department of Oncology, Zhujiang Hospital, Southern Medical University, Guangzhou, 510280, China; [zhangjian@i.smu.edu.cn](mailto:zhangjian@i.smu.edu.cn)  Wen Huang; Department of Oncology, Zhujiang Hospital, Southern Medical University, Guangzhou, 510280, China; w_huang@i.smu.edu.cn |
| --- | --- |
| **Sub Investigators** | Junyi Shen; Department of Oncology, Zhujiang Hospital, Southern Medical University, Guangzhou, 510280, China; sjy@i.smu.edu.cn |
| **Funding Sponsor:** | Not applicable |

**Table of Contents**

1. Supplementary Table 1…………………………………………..........................................3
2. Supplementary Table 2………………………………………….........................................18
3. Supplementary Table 3………………………………………….........................................32
4. Supplementary Table 4………………………………………….........................................36
5. Supplementary Table 5………………………………………….........................................37
6. Supplementary Table 6………………………………………….........................................38

7. Supplementary Figure 1…………………………………………........................................39

8. Supplementary Figure 2…………………………………………........................................40

**Supplementary Table 1. Generic and trade names of anthracyclines and their derivatives included in the pharmacovigilance analysis of coagulation-related adverse events.**

| aclacinomycin (aclarubicin) unspecified | aclarubicin | aclarubicin [aclarubicin hydrochloride] | aclarubicin hcl |
| --- | --- | --- | --- |
| aclarubicin hydrochloride | aclarubicine | aclacinomycin | aclacinomycin a |
| aclacinomycin hydrochloride | aclacinon | aclacin | aldoxorubicin |
| amrubicin | amrubicin hydrochloride | amrubicinum | calsed |
| daunorubicin | daunorubicin hydrochloride | hydroxydaunorubicin | daunorubicin hcl |
| daunorubicine | (daunorubicin) | ade [daunorubicin] | blinded daunorubicin hydrochloride |
| chlorhydrate de daunorubicine | comparator daunorubicin | danuicin (daunorubicin hydrochloride) | daunomycin (daunorubicin hydrochloride) |
| daunomycin (daunorubicin) (unknown) (daunorubicin) | daunomycin [daunorubicin hydrochloride] | daunomycin [daunorubicin] | daunomycin//daunorubicin hydrochloride |
| daunorubicin /00128202/ | daunorubicin (384mg) | daunorubicin (baunorubicin) (daunorubicin) | daunorubicin (danorubicin) |
| daunorubicin (daunorubicin hydrochloride) | daunorubicin (daunorubicin hydrochloride) infusion | daunorubicin (daunorubicin) | daunorubicin (daunorubicin) (daunorubicin ) |
| daunorubicin (daunorubicin) (daunorubicin) | daunorubicin (daunorubicin) (unknown) | daunorubicin (daunorubicin0 (daunorubicin) | daunorubicin (dnr) |
| daunorubicin [daunorubicin hydrochloride] | daunorubicin equivalents | daunorubicin hcl (daunorubicin hydrochloride) | daunorubicin hcl inj. 20mg/4ml - bedford labs, inc. |
| daunorubicin hcl injection (daunorubicin) | daunorubicin hcl, 20mg/4ml | daunorubicin hydrochloride (daunorubicin) (unknown) (daunorubicin) | daunorubicin hydrochloride for injection |
| daunorubicin hydrochloride. | daunorubicin lens | daunorubicin liposomal | daunorubicin-lans |
| daunorubicin(daunorubicin)(daunorubicin) | daunorubicina | daunorubicina /00128201/ | daunorubicina hidrocloruro (177ch) |
| daunorubicine (daunorubicin) | daunorubicine (daunorubicine) | daunorubicine dci | daunorubicine pdr v infvlst 20mg |
| daunorubicinhydrochloride for injection | daunorubicinum | daunoxome [daunorubicin hydrochloride] | external-daunorubicin |
| liposomal daunorubicin | low dose daunorubicin | tn unspecified (daunorubicin) | tn unspecified [daunorubicin hydrochloride] |
| tn unspecified [daunorubicin] | cerubidine | cerubidine 20 mg | cerubidine, daunoxome |
| daunoxome | daunomycin | daunomycin /00128201/ | daunomycin /00128202/ |
| daunomycin (daunomycin) | daunomycin /00128201/ | daunomycin hcl | daunomycin hydrochloride |
| rubidomycin | hydroxy daunorubicin | hydroxydaunorubicin hydrochloride | hydroxydaunorubicine |
| doxorubicin | doxorubicin hydrochloride | doxorubicin liposomal | doxil |
| doxorubicin hcl | doxorubicine | doxorubicin hydrochloride liposome injection 20 mg | doxorubicin hydrochloride liposome injection |
| pegylated liposomal doxorubicin | doxorubicin hydrochloride injection | liposomal doxorubicin | doxorubicine teva |
| pegylated liposomal doxorubicin hydrochloride | liposomal doxorubicin hydrochloride | adriamycin [doxorubicin] | doxorubicine [doxorubicin] |
| liposomal doxorubicin hcl | doxorubicin hydrochloride liposome | doxorubicine accord | (doxorubicin |
| (doxorubicin ) | (doxorubicin hydrochloride) | (doxorubicin) | (doxorubicine /00330902/) |
| actavis standard doxorubicin | adriamycin ( doxorubicin) ( doxorubicin) | adriamycin (doxorubicin) | adriamycin (doxorubicin) (doxorubicin) |
| adriamycin (doxorubicin) inj. usp 10mg/5ml | adriamycin (doxorubicin) inj. usp 10mg/5ml - bedford labs, inc. | adriamycin [doxorubicin hydrochloride] | adriamycin//doxorubicin |
| adricin (doxorubicin hydrochloride) | adricin (doxorubicin) | amycin (doxorubicin) | andriamycin(doxorubicin)(doxorubicin) |
| blinded doxorubicin | caelyx (doxorubicin hydrochloride) | caelyx (doxorubicin hydrochloride) | caelyx (liposomal doxorubicin) |
| caelyx (pegylated liposomal doxorubicin) | chlorhydrate de doxorubicine | dc; drug?eluting bead doxorubicin | doxil (doxorubicin hydrochloride) |
| doxil (doxorubicin hydrochloride) | doxil (doxorubicin hydrochloride) (unknown) | doxil (doxorubicine hydrochloride) | doxil (pegylated liposomal doxorubicin hydrochloride) |
| doxil [pegylated liposomal doxorubicin hydrochloride] | doxil, doxorubicin hydrochloride | doxin [doxorubicin hydrochloride] | doxlox (doxorubicin) |
| doxoeubicin (doxorubicin) | doxolex [doxorubicin] | doxor [doxorubicin hydrochloride] | doxorubicin /00330901/ |
| doxorubicin /00330902/ | doxorubicin /00330902/ | doxorubicin hydrochloride | doxorubicin hydrochloride injection |
| doxorubicin hydrochloride injection, usp | doxorubicin teva 2 mg/ml | doxorubicin ( doxorubicin) ( doxorubicin) | doxorubicin (1 cycle) |
| doxorubicin (202a) | doxorubicin (doxorubicin hydrochloride) | doxorubicin (doxorubicin hydrochloride) unknown | doxorubicin (doxorubicin) |
| doxorubicin (doxorubicin) (doxorubicin) | doxorubicin (doxorubicin) (unknown) (doxorubicin) | doxorubicin (doxorubicin) injection | doxorubicin (doxorubicin)(unknown)(doxorubicin) |
| doxorubicin (doxorubicin/doxorubicin hydrochloride, doxorubicin) | doxorubicin (doxorubicin/doxorubidin hydrochloride) | doxorubicin (doxorubiin) | doxorubicin (manufacturer unknown) |
| doxorubicin (manufacturer unknown) (doxoroubicin) | doxorubicin (manufacturer unknown) (doxorubicin hdyrochloride) (doxorubicin hydrochloride) | doxorubicin (manufacturer unknown) (doxorubicin hydrochloride) (doxorubcin hydrochloride) | doxorubicin (manufacturer unknown) (doxorubicin hydrochloride) (doxorubicin hydorchloride) ? |
| doxorubicin (manufacturer unknown) (doxorubicin hydrochloride) (doxorubicin hydrochloride) | doxorubicin (manufacturer unknown) (doxorubicin) (doxorubicin) | doxorubicin (open label) | doxorubicin (unknown) |
| doxorubicin [doxorubicin hydrochloride] | doxorubicin /00330901/ | doxorubicin /00330902/ | doxorubicin /00330902/ (doxorubicin hydrochloride) |
| doxorubicin #1 /00330902/ | doxorubicin ^teva^ | doxorubicin 200mg/100ml | doxorubicin 25 mg/m2 iv days 1?2, repeat q21d x4 cycles |
| doxorubicin 25 mg/m2 iv days 1?2, repeat q21d x5 cycles | doxorubicin 2mg/ml | doxorubicin 37.5 mg/m2 iv x1?2 days every 4 weeks | doxorubicin 60mg/250ml |
| doxorubicin accord | doxorubicin aurovitas | doxorubicin concentrate for solution for infusion | doxorubicin ebewe |
| doxorubicin ebewe 2 mg/ml | doxorubicin hci liposome injection | doxorubicin hcl (doxorubicin hydrochloride) | doxorubicin hcl injection-sagent |
| doxorubicin hcl liposomal | doxorubicin hcl liposome | doxorubicin hcl liposome, 40mg/m2 | doxorubicin hcl peg-liposomal 2 mg/ml |
| doxorubicin hcl sdv 2mg/ml pfizer u.s. | doxorubicin hcl(doxorubicin hydrochloride) | doxorubicin hexal | doxorubicin hydrichloride |
| doxorubicin hydrochhloride | doxorubicin hydrochloride injection | doxorubicin hydrochloride (actavis inc) | doxorubicin hydrochloride (actavis llc) |
| doxorubicin hydrochloride (caelyx) | doxorubicin hydrochloride (doxil | doxorubicin hydrochloride (doxil) | doxorubicin hydrochloride (doxorubicin hydrochloride) |
| doxorubicin hydrochloride (pegylated liposomal) | doxorubicin hydrochloride 50mg [nk] | doxorubicin hydrochloride for injection | doxorubicin hydrochloride for injection 50mg |
| doxorubicin hydrochloride injection 2mg/ml | doxorubicin hydrochloride injection, usp | doxorubicin hydrochloride injection: | doxorubicin hydrochloride liposomal |
| doxorubicin hydrochloride liposome injection 20 mg/10 ml (2 mg/ml) and | doxorubicin hydrochloride liposome injection usp 2mg/ml | doxorubicin hydrochloride nk | doxorubicin hydrochloride sandoz |
| doxorubicin hydrochloride solution for infusion | doxorubicin hydrochloride solution for injection 2 mg/ml | doxorubicin hydrochloride unspecified | doxorubicin hydrochloride, liposomal |
| doxorubicin hydrochloride: | doxorubicin hydrochloride? | doxorubicin hydrochloride. | doxorubicin hydrochloride(caelyx) |
| doxorubicin hydrochloride(doxil) | doxorubicin hydrochloride(doxorubicin hydrochloride) | doxorubicin hydrocholoride injection | doxorubicin hydrocloride |
| doxorubicin hydrpchloride injection | doxorubicin hyrochloride | doxorubicin inj 10/5ml | doxorubicin inj 10/5ml, |
| doxorubicin inj 10mg | doxorubicin inj 2mg/ml | doxorubicin inj 50/25ml | doxorubicin injectie/infuus |
| doxorubicin injection | doxorubicin iv | doxorubicin lipo | doxorubicin lipo 2mg/ml |
| doxorubicin liposome | doxorubicin mylan | doxorubicin nos | doxorubicin nos (open label) |
| doxorubicin pf + sodium chloride | doxorubicin pfizer | doxorubicin pliva | doxorubicin powder for solution for infusion |
| doxorubicin sandoz | doxorubicin solution for injection/infusion | doxorubicin suspension for injection | doxorubicin teva |
| doxorubicin teva 2 mg/ml | doxorubicin teva 50 mg/25 ml | doxorubicin-teva | doxorubicin, 20mg/10ml pfizer |
| doxorubicin, 50mg/25ml fresnius kabi | doxorubicin, 79.6 mg | doxorubicin?ebewe | doxorubicin?liposomal |
| doxorubicin?pfizer | doxorubicin(doxorubicin) | doxorubicin/doxorubicin hydrochloride | doxorubicin/unknown/unknown |
| doxorubicina | doxorubicina /00330901/ | doxorubicina (202a) | doxorubicina [doxorubicin hydrochloride] |
| doxorubicina [doxorubicin] | doxorubicina accord | doxorubicina accord healthcare italia | doxorubicina accord healthcare italia - 2mg/ml |
| doxorubicina aurobindo | doxorubicina cloridrato | doxorubicina cloroidrato | doxorubicina ebewe |
| doxorubicina pfizer | doxorubicina sandoz | doxorubicina solution for infusion, 2 mg/ml | doxorubicina teva |
| doxorubicina teva - 2 mg/ml concentrato per soluzione per infusione | doxorubicina teva - teva italia s.r.l. | doxorubicina teva 2 mg/ml concentrato per soluzione per infusione | doxorubicine /00330901/ |
| doxorubicine /00330902/ | doxorubicine hydrochloride | doxorubicine (chlorhydrate ) | doxorubicine (chlorhydrate de) |
| doxorubicine (doxorubicin hydrochloride) | doxorubicine /00330901/ | doxorubicine accord 2 mg/ml | doxorubicine accord 2 mg/ml, solution pour perfusion |
| doxorubicine arrow | doxorubicine chlorhydrate | doxorubicine chlorhydrate teva | doxorubicine dakota pharm |
| doxorubicine ebewe | doxorubicine ev | doxorubicine hydrochloride | doxorubicine in liposomes study drug |
| doxorubicine infuus (liposomaal) | doxorubicine injvlst 2mg/ml | doxorubicine teva 200 mg/100 ml | doxorubicine teva 200 mg/100 ml, solution injectable |
| doxorubicine teva 200mg/100ml | doxorubicine teva 50 mg/25 ml | doxorubicine teva 50 mg/25 ml, solution injectable | doxorubicine teva 50mg/25ml |
| doxorubicinhydrochlorid teva 2 mg/ml konzentrat zur herstellung einer | doxorubicinum accord | external-doxorubicin hydrochloride | hydroxy-doxorubicin |
| hydroxydoxorubicin | kai lai [doxorubicin hydrochloride] | lipo-doxorubicin | liposomal doxorubicin (doxorubicin hydrochloride) |
| liposomal doxorubicin (myocet) | liposomal doxorubicin jannsen | liposomal doxorubicine | liposomal encapsulated doxorubicin |
| liposomal formulation of doxorubicin | liposomal-doxorubicin | lixing [pegylated liposomal doxorubicin hydrochloride] | myocet - teva b.v. (liposomal doxorubicin hydrochloride) |
| non pegylated liposomal doxorubicin | non pegylated liposome encapsulated doxorubicin | non-pegylated liposomal doxorubicin | non-pegylated liposomal doxorubicin hydrochl |
| non-pegylated liposomal doxorubicin hydrochl. | non-pegylated liposome-encapsulated doxorubicin | nonpegylated liposomal doxorubicin | peglylated liposomal doxorubicin |
| pegylated doxorubicin | pegylated doxorubicin hydrochloride | pegylated liposoma doxorubicin hydrochloride | pegylated liposomal doxorubicin hcl |
| pegylated liposomal doxorubicin hydrochloride(pegylated liposomal doxo | pfizer doxorubicin | previous chemotherapy with liposomal doxorubicin | rubicin [doxorubicin hydrochloride] |
| rubidox (doxorubicin hydrochloride) | teva doxorubicin | tn unspecified [doxorubicin hydrochloride] | tn unspecified [doxorubicin] |
| 14-hydroxydaunomycin hcl | 14-hydroxydaunomycin hydrochloride | hydroxydaunomycin | hydroxydaunomycin hydrochloride |
| adriamycin | adriamycin /00330901/ | adriamycin (adriamycin) | adriamycin /0030901/ |
| adriamycin /00330901/ | adriamycin cs | adriamycin hcl | adriamycin hs |
| adriamycin hydrochloride | adriamycin inj | adriamycin rdf | adriamycin, doxil |
| adriamycin/00330901/ | liposomal adriamycin | pegylated liposome adriamycin | doxil /00055703/ |
| doxil /00330902/ | doxil (israel) | doxil (japan) | doxil (nos) |
| doxil (united states) | doxil (unk ingredients) | doxil /00330902/ | doxil /00330904/ |
| doxil 30mg/m2 | doxil glucosamine | (caelyx) | caelyx |
| caelyx - 2 mg/ml concentrato per soluzione per infusione | caelyx 2 mg/ml | caelyx 2 mg/ml concentrate for solution for infusion | caelyx 2 mg/ml, solution ? diluer pour perfusion |
| caelyx 2 mg/ml, solution a diluer pour perfusion | caelyx 20 mg/10 ml | caelyx au | caelyx injection |
| caelyx pegylated liposomal 2 mg/ml concentrate for solution for infusi | caelyx q28 | caelyx single use vials. 20mg/10ml and 50mg/25ml. peg. liposomal suspe | caelyx single use vials. 20mg/10ml and 50mg/25ml. peg.liposomalsuspens |
| pegylated liposomal dexorubicin | myocet | myocet /00330902/ | myocet /00330903/ |
| myocet - 50 mg polvere e additivi per concentrato per dispersione lipo | myocet ? teva b.v. | myocet /00330903/ | myocet 50 mg polvo y premezclas concentrado para dispersion liposomica |
| myocet 50 mg powder, dispersion and solvent for concentrate for disper | myocet 50mg polvo y premezclas concentrado para dispersion liposomica | epidoxorubicin | epidoxorubicina |
| pms?epirubicin | (epirubicin) | ai da sheng (epirubicin) | ai da sheng [epirubicin hydrochloride] |
| chlorhydrate d'epirubicine | epirubicin | epirubicin /00699302/ | epirubicin hydrochloride |
| epirubicin - 50mg/25ml (mfr: sagent) | epirubicin -unk mah | epirubicin (epirubicin hydrochloride) | epirubicin (epirubicin hydrochloride) injection |
| epirubicin (epirubicin hydrochloride) injection, 110mg/m2 | epirubicin (epirubicin) | epirubicin (epirubicin) (epirubicin) | epirubicin (epirubicin) (solution for infusion) (epirubicin) |
| epirubicin (manufacturer unknown) | epirubicin (manufacturer unknown) (epirubicin hydrochloride) (epirubicin hydrochloride) | epirubicin (unknown) | epirubicin (unknown) (epirubicin hydrochloride) unk, unkunk |
| epirubicin [nk] (epirubicin hydrochloride) | epirubicin /00699302/ | epirubicin ^actavis^ | epirubicin ^ebewe^ |
| epirubicin ^hospira^ | epirubicin ^teva^ | epirubicin accord | epirubicin actavis |
| epirubicin ahcl | epirubicin e (manufacturer unknown) (epirubicin hydrochloride)epirubicin hydrochloride) (epirubicin hydrochloride) | epirubicin ebewe | epirubicin hcl |
| epirubicin hexal | epirubicin hospira | epirubicin hydrochloride | epirubicin hydrochloride (actavis) |
| epirubicin hydrochloride (atllc) | epirubicin hydrochloride (unknown) | epirubicin hydrochloride accord | epirubicin hydrochloride inj. 10mg ^nk^ |
| epirubicin hydrochloride inj. 50mg ^nk^ | epirubicin hydrochloride injection | epirubicin hydrochloride injection 10mg ^nk^ | epirubicin hydrochloride injection 10mg/5ml ^mylan^ |
| epirubicin hydrochloride injection 50mg ^nk^ | epirubicin hydrochloride mylan | epirubicin hydrochloride pch | epirubicin hydrochloride. |
| epirubicin medac | epirubicin mylan | epirubicin pms | epirubicin sandoz |
| epirubicin solution for injection | epirubicin teva | epirubicina | epirubicina (364a) |
| epirubicina /00699301/ | epirubicina ahcl | epirubicina cloridrato | epirubicina cloroidrato |
| epirubicina hospira | epirubicina mylan | epirubicina teva | epirubicina teva - 2 mg/ml soluzione iniettabile o per infusione |
| epirubicina teva 2 mg/ml solucion inyectable y para perfusion efg | epirubicina teva 2 mg/ml soluzione iniettabile o per infusione | epirubicina teva 200 mg/100 ml | epirubicine |
| epirubicine (chlorhydrate d') | epirubicine actavis | epirubicine ebewe | epirubicine infusiepoeder 10mg |
| epirubicine infuus | epirubicine injectie/infuus / brand name not specified | epirubicine medac | epirubicine mylan |
| epirubicine mylan 2 mg/ml | epirubicine mylan 2 mg/ml solution pour perfusion | epirubicine mylan 2 mg/ml, solution for infusion | epirubicine mylan 2 mg/ml, solution pour perfusion |
| epirubicine sandoz | epirubicine teva | epirubicine teva 2 mg/ml, solution pour perfusion | epirubicine teva classics |
| epirubicine winthrop | epirubicini hydrochloridum | epirubicinijev klorid pch 2 mg/ml | epirubicinijev klorid pch 2 mg/ml raztopina za injiciranje ali infundi |
| fa ma xin [epirubicin hydrochloride] | famaxin (epirubicin hydrochloride) | famaxine (epirubicin hydrochloride) | farmaxin (epirubicin hydrochloride) |
| forced [epirubicin hydrochloride] | ive [epirubicin] | pms-epirubicin | pms? epirubicin |
| terry (epirubicin hydrochloride) | epiadriamycin | ellence | farmorubicin (pharmorubicin) |
| pharmorubicin | pharmorubicin rd | pharmorubicin?rd | (farmorubicina) |
| farmorubicin | farmorubicin 10mg | farmorubicin rd | farmorubicin rtu |
| farmorubicina | farmorubicina - pfizer limited | farmorubicina pfizer | farmorubicine |
| idarubicin hydrochloride | blinded idarubicin | chlorhydrate d'idarubicine | comparator idarubicin |
| external-idarubicin | idarubicin | idarubicin /00830302/ | idarubicin (ida) |
| idarubicin (idarubicin) | idarubicin (idarubicin) (idarubicin) | idarubicin /00830302/ | idarubicin comp_ida+ |
| idarubicin hcl | idarubicin hydrochloride (idarubicin hcl) | idarubicin hydrochloride (idarubicin hydrochloride) | idarubicin hydrochloride (manufacturer unknown) |
| idarubicin hydrochloride (manufacturer unknown) (idarubicin hydrochloride) (idarubicin hydrochloride) | idarubicin hydrochloride. | idarubicin hydrochoride | idarubicin mylan |
| idarubicin pfizer | idarubicin/idarubicin hydrochloride | idarubicina | idarubicina /00830301/ |
| idarubicina (2550a) | idarubicina [idarubicin hydrochloride] | idarubicina [idarubicin] | idarubicina /00830301/ |
| idarubicina hidrocloruro (2550ch) | idarubicine | idarubicine (chlorhydrate d') | idarubicine mylan |
| zavedos (idarubicin hydrochloride) | idamycin | zavedos | zavedos - 10 mg/10 ml soluzione iniettabile - pfizer italia s.r.l. |
| zavedos - pfizer italia s.r.l. | zavedos 10 mg/10 ml soluzione iniettabile per uso endovenoso | 4-0-tetrahydropyranyladriamycin (doxorubicin) (doxorubicin) | 4-o tetrahydropyranyladriamycin (doxorubicin) (doxorubicin) |
| tetrahydropyranyl doxorubicin | pinorubin [pirarubicin hydrochloride] | pinorubin [pirarubicin] | pirarubicin |
| pirarubicin (pirarubicin) | pirarubicin (pirarubicin) (pira-rubicin) | pirarubicin (pirarubicin) (pirarubicin) | pirarubicin /00963102/ |
| pirarubicin hcl | pirarubicin hydrochloride | pirarubicin mixed emulsion | pirarubicin/pirarubicin hydrochloride |
| pirarubicine | therarubicin [pirarubicin hydrochloride] | yibilu (pirarubicin hydrochloride) | 49-0-tetrahydropyranyladriamycin |
| tetrahydropyranyl adriamycin | tetrahydropyranyladriamycin | thp-adriamycin | thp?adriamycin |
| therarubicin | therarubicin /00963102/ | therarubicin /00963101/ | thp |
| theprubicine | pinorubin | pinorubin /00963102/ | pinorubin /00963102/ |
| valstar |  |  |  |

**Supplementary Table 2. Search terms for coagulation-related adverse reaction PTs in MedDRA 25.1.**

| Coagulation test abnormal | Fibrin | Factor X deficiency | Heparin-induced thrombocytopenia test positive |
| --- | --- | --- | --- |
| Platelet function test abnormal | Fibrin abnormal | Arteriovenous graft thrombosis | Cerebral septic infarct |
| Platelet disorder | Fibrin D dimer | Circulating anticoagulant | Von Willebrand's factor inhibition |
| Hypofibrinogenaemia | Fibrin D dimer increased | Acquired haemophilia | Cerebral gas embolism |
| Coagulation factor deficiency | Fibrin D dimer decreased | Haemophilia A with anti factor VIII | Congenital hypercoagulation |
| Thrombasthenia | Fibrin D dimer normal | Haemophilia B with anti factor IX | Platelet glycoprotein gene mutation |
| Activated partial thromboplastin time | Fibrin decreased | Haemophilia A without inhibitors | Plasminogen activator inhibitor polymorphism |
| Activated partial thromboplastin time abnormal | Fibrin degradation products increased | Haemophilia B without inhibitors | Haemorrhagic vasculitis |
| Activated partial thromboplastin time shortened | Fibrin degradation products normal | White clot in blood present | Spinal artery thrombosis |
| Activated partial thromboplastin time prolonged | Fibrin increased | Thrombin-antithrombin III complex increased | Anti-thrombin antibody |
| Activated partial thromboplastin time normal | Fibrin normal | Thrombin-antithrombin III complex decreased | Umbilical cord thrombosis |
| Pulmonary embolism | Plasmin decreased | Thrombin-antithrombin III complex | Thrombocytopenia-absent radius syndrome |
| Platelet adhesiveness abnormal | Plasmin increased | Thrombin-antithrombin III complex normal | Hermansky-Pudlak syndrome |
| Factor I deficiency | Fibrinolysis | Thrombin-antithrombin III complex abnormal | Ovarian vein thrombosis |
| Platelet aggregation abnormal | Fibrinolysis abnormal | Mean platelet volume increased | Endothelial protein C receptor polymorphism |
| Wiskott-Aldrich syndrome | Fibrinolysis decreased | Mean platelet volume decreased | Renal vascular thrombosis |
| Plasmin inhibitor decreased | Fibrinolysis increased | Mean platelet volume normal | Foetal vascular malperfusion |
| Plasmin inhibitor increased | Fibrinolysis normal | Mean platelet volume abnormal | Post transfusion purpura |
| Anaphylactoid syndrome of pregnancy | Hypocoagulable state | Arteriovenous graft site stenosis | Anti factor X antibody negative |
| Cerebral artery embolism | Glanzmann's disease | Von Willebrand's factor multimers abnormal | Non-immune heparin associated thrombocytopenia |
| Cerebral artery thrombosis | Haemorrhagic disorder | Von Willebrand's factor multimers test | Anti factor X antibody |
| Coagulation time shortened | Haemorrhagic disease of newborn | Von Willebrand's factor multimers normal | Prekallikrein test normal |
| Coagulation time prolonged | Hepatic artery embolism | Factor V inhibition | Prekallikrein test abnormal |
| Coagulation factor VIII level | Hepatic artery thrombosis | Activated protein C resistance | Prekallikrein increased |
| Antiphospholipid syndrome | Hepatic vein thrombosis | Bernard-Soulier syndrome | Prekallikrein decreased |
| Antithrombin III deficiency | Hypercoagulation | Blue toe syndrome | Septic vasculitis |
| Aortic embolus | Pulmonary infarction | Vertebral artery thrombosis | Soluble fibrin monomer complex increased |
| Aortic thrombosis | Immune thrombocytopenia | Infective thrombosis | Platelet dysfunction |
| Peripheral embolism | Platelet count increased | Hyperthrombinaemia | Instillation site thrombosis |
| Embolism arterial | Increased tendency to bruise | Hypothrombinaemia | Microembolism |
| Arterial thrombosis | Infantile scurvy | Hypothromboplastinaemia | Haemophilic pseudotumour |
| Peripheral artery thrombosis | Vena cava thrombosis | Embolia cutis medicamentosa | Ophthalmic vein thrombosis |
| Arteriovenous fistula thrombosis | Injection site thrombosis | Anti factor V antibody positive | Brain stem embolism |
| Vitamin C deficiency | International normalised ratio increased | Anti factor VII antibody positive | ADAMTS13 activity increased |
| Antithrombin III decreased | International normalised ratio abnormal | Anti factor X antibody positive | ADAMTS13 activity decreased |
| Platelet morphology abnormal | International normalised ratio normal | Anti factor IX antibody positive | ADAMTS13 activity normal |
| Axillary vein thrombosis | Cerebral venous sinus thrombosis | Anti factor XI antibody positive | ADAMTS13 activity abnormal |
| International normalised ratio | Jugular vein thrombosis | Anti factor XII antibody positive | Hepatic vascular thrombosis |
| Haemorrhagic diathesis | Platelet count decreased | Congenital thrombocyte disorder | Stoma site thrombosis |
| Bleeding time | Venous thrombosis limb | Coronary bypass thrombosis | Spontaneous haemorrhage |
| Bleeding time shortened | Mesenteric artery thrombosis | Cullen's sign | Acquired antithrombin III deficiency |
| Bleeding time prolonged | Thrombosis mesenteric vessel | Shunt thrombosis | Splenic artery thrombosis |
| Bleeding time normal | Mesenteric vein thrombosis | Hoigne's syndrome | Splenic thrombosis |
| Blood fibrinogen | Moyamoya disease | Platelet toxicity | Precerebral artery thrombosis |
| Blood fibrinogen abnormal | Obstetrical pulmonary embolism | Heparin resistance | Thromboelastogram |
| Blood fibrinogen decreased | Paroxysmal nocturnal haemoglobinuria | Factor XIII inhibition | Hyperfibrinolysis |
| Blood fibrinogen increased | Vascular purpura | Plasminogen activator inhibitor increased | Postsplenectomy syndrome |
| Blood fibrinogen normal | Pelvic venous thrombosis | Plasminogen activator inhibitor decreased | Neonatal alloimmune thrombocytopenia |
| Blood thrombin | Penile vein thrombosis | Plasminogen activator inhibitor | Adrenal thrombosis |
| Blood thrombin abnormal | Petechiae | Coagulation factor | Factor VII inhibition |
| Blood thrombin decreased | Superficial vein thrombosis | Russell's viper venom time normal | Factor X inhibition |
| Blood thrombin increased | Venous thrombosis | Russell's viper venom time abnormal | Factor II inhibition |
| Blood thrombin normal | Plasminogen increased | Russell's viper venom time | Foreign body embolism |
| Blood thromboplastin | Plasminogen decreased | Platelet morphology | Activated partial thromboplastin time ratio |
| Blood thromboplastin abnormal | Plasminogen normal | Plasmin inhibitor | Activated partial thromboplastin time ratio abnormal |
| Blood thromboplastin decreased | Platelet adhesiveness decreased | Platelet factor 4 | Activated partial thromboplastin time ratio decreased |
| Blood thromboplastin increased | Platelet adhesiveness increased | Soluble fibrin monomer complex | Activated partial thromboplastin time ratio fluctuation |
| Blood thromboplastin normal | Platelet aggregation decreased | Platelet factor 4 increased | Activated partial thromboplastin time ratio increased |
| Budd-Chiari syndrome | Platelet aggregation increased | Platelet factor 4 decreased | Activated partial thromboplastin time ratio normal |
| Capillary fragility abnormal | Platelet aggregation normal | Platelet morphology normal | Anti factor IX antibody |
| Capillary fragility increased | Platelet count | Protein C increased | Grey Turner's sign |
| Capillary fragility normal | Platelet count abnormal | Plasminogen | Mahler sign |
| Capillary permeability | Platelet count normal | Embolic cerebral infarction | Administration site thrombosis |
| Capillary permeability increased | Platelet function test normal | Dilutional coagulopathy | Application site thrombosis |
| Capillary permeability normal | Platelet production decreased | Platelet function test | Medical device site thrombosis |
| Capillary fragility test | Portal pyaemia | Platelet adhesiveness | Vaccination site thrombosis |
| Capillary fragility decreased | Portal vein thrombosis | Platelet adhesiveness normal | Anti factor V antibody |
| Carotid arterial embolus | Post thrombotic syndrome | Truncus coeliacus thrombosis | Amegakaryocytic thrombocytopenia |
| Carotid artery thrombosis | Thrombocytopenia | Brain stem thrombosis | Acquired amegakaryocytic thrombocytopenia |
| Cavernous sinus thrombosis | Prothrombin time | Ethanol gelation test positive | ADAMTS13 inhibitor screen assay |
| Retinal vein occlusion | Chronic pigmented purpura | Ethanol gelation test negative | ADAMTS13 activity assay |
| Cerebellar artery thrombosis | Protein C decreased | Ethanol gelation test | Anti factor VIII antibody increased |
| Cerebral thrombosis | Prothrombin time ratio | Prekallikrein test | Anti factor IX antibody increased |
| Cerebral venous thrombosis | Prothrombin time ratio decreased | Coagulation factor inhibitor assay | Postpartum thrombosis |
| Atheroembolism | Prothrombin time abnormal | Platelet storage pool deficiency | Portosplenomesenteric venous thrombosis |
| Factor IX deficiency | Prothrombin time shortened | Basilar artery thrombosis | Anti factor X activity |
| Coagulation factor IX level | Prothrombin time prolonged | Prosthetic cardiac valve thrombosis | Anti factor X activity normal |
| Thrombosis | Prothrombin time normal | Coagulation factor X level normal | Anti factor X activity abnormal |
| Clot retraction | Prothrombin time ratio abnormal | Coagulation factor XI level normal | Anti factor X activity increased |
| Clot retraction abnormal | Prothrombin time ratio increased | Coagulation factor XII level normal | Anti factor X activity decreased |
| Clot retraction time shortened | Pulmonary artery thrombosis | Coagulation factor XIII level normal | Factor Xa activity test |
| Clot retraction normal | Pulmonary microemboli | Prothrombin consumption time prolonged | Visceral venous thrombosis |
| Clot retraction time prolonged | Pulmonary thrombosis | Prothrombin consumption time shortened | Congenital thrombocytopenia |
| Coagulopathy | Pulmonary veno-occlusive disease | International normalised ratio fluctuation | Calcium embolism |
| Coagulation time | Pulmonary venous thrombosis | Brachiocephalic vein thrombosis | Vascular pseudoaneurysm thrombosis |
| Coagulation disorder neonatal | Purpura fulminans | Hypothenar hammer syndrome | Cerebral microembolism |
| Coagulation factor decreased | Purpura neonatal | Coagulation test | Severe fever with thrombocytopenia syndrome |
| Prothrombin level | Purpura senile | Coagulation test normal | Factor Xa activity abnormal |
| Prothrombin level abnormal | Thrombocytopenic purpura | Congenital coagulopathy | Factor Xa activity decreased |
| Prothrombin level decreased | Thrombotic thrombocytopenic purpura | Circulating anticoagulant positive | Factor Xa activity increased |
| Prothrombin level increased | Renal embolism | Vessel puncture site thrombosis | Factor Xa activity normal |
| Prothrombin level normal | Renal artery thrombosis | Implant site thrombosis | Hepatic vein embolism |
| Coagulation factor increased | Renal vein thrombosis | Post procedural pulmonary embolism | Gardner-Diamond syndrome |
| Coagulation factor IX level abnormal | Retinal artery thrombosis | Anti factor VIII antibody negative | Anti factor Xa activity decreased |
| Coagulation factor IX level decreased | Retinal vein thrombosis | Anti factor IX antibody negative | Paraneoplastic thrombosis |
| Coagulation factor IX level increased | Superior sagittal sinus thrombosis | Vascular stent thrombosis | Catheter site thrombosis |
| Coagulation factor IX level normal | Thrombophlebitis septic | Venous thrombosis neonatal | Achenbach syndrome |
| Coagulation factor level normal | Splenic vein thrombosis | Thromboxane increased | Anti factor Xa assay normal |
| Coagulation factor V level | Subclavian artery embolism | Thromboxane decreased | Pulmonary tumour thrombotic microangiopathy |
| Coagulation factor V level abnormal | Subclavian artery thrombosis | Plateletcrit | Fat embolism syndrome |
| Coagulation factor V level decreased | Thrombin time | Plateletcrit increased | Von Willebrand's factor antibody |
| Coagulation factor V level increased | Thrombin time normal | Plateletcrit decreased | Spontaneous heparin-induced thrombocytopenia syndrome |
| Coagulation factor V level normal | Thrombin time prolonged | Plateletcrit abnormal | Ophthalmic artery thrombosis |
| Coagulation factor VII level | Thrombin time shortened | Plateletcrit normal | Jugular vein embolism |
| Coagulation factor VII level abnormal | Thrombocytosis | Platelet distribution width increased | Portal vein embolism |
| Coagulation factor VII level decreased | Thrombocytopenia neonatal | Platelet distribution width decreased | Prothrombin fragment 1.2 |
| Coagulation factor VII level increased | Thrombophlebitis | Factor II mutation | Plasminogen activator inhibitor type 1 deficiency |
| Coagulation factor VII level normal | Thrombophlebitis migrans | Spontaneous haematoma | Acquired factor VIII deficiency |
| Coagulation factor VIII level abnormal | Thrombophlebitis neonatal | Von Willebrand's factor antigen increased | Acquired factor XI deficiency |
| Coagulation factor VIII level decreased | Thrombotic microangiopathy | Coagulation factor mutation | Acquired factor IX deficiency |
| Coagulation factor VIII level increased | Thrombotic stroke | MYH9-related disease | Peripheral vein thrombus extension |
| Coagulation factor VIII level normal | Transverse sinus thrombosis | Infusion site thrombosis | Antithrombin III abnormal |
| Coagulation factor X level | Tumour embolism | Lemierre syndrome | Prothrombin fragment 1.2 increased |
| Coagulation factor X level abnormal | Cardiac ventricular thrombosis | Embolic pneumonia | Septic pulmonary embolism |
| Coagulation factor X level decreased | Factor VIII inhibition | Paradoxical embolism | Secondary thrombocytosis |
| Coagulation factor X level increased | Intracardiac thrombus | Intracardiac mass | Septic coagulopathy |
| Coagulation factor XI level | Atrial thrombosis | Platelet anisocytosis | Lambl's excrescences |
| Coagulation factor XI level abnormal | Heparin-induced thrombocytopenia | Von Willebrand's factor antibody positive | Autoimmune heparin-induced thrombocytopenia |
| Coagulation factor XI level decreased | Factor V deficiency | Deep vein thrombosis postoperative | Embolic cerebellar infarction |
| Coagulation factor XI level increased | Anti factor VIII antibody positive | Device related thrombosis | Prothrombin index |
| Coagulation factor XII level | HELLP syndrome | Cerebellar embolism | Post thrombotic retinopathy |
| Coagulation factor XII level abnormal | Bleeding time abnormal | Thrombosis corpora cavernosa | Aseptic cavernous sinus thrombosis |
| Coagulation factor XII level decreased | Retinal vascular thrombosis | Thrombotic cerebral infarction | Lupus anticoagulant hypoprothrombinaemia syndrome |
| Coagulation factor XII level increased | Anti-prothrombin antibody positive | Activated protein C resistance test | Precerebral artery embolism |
| Coagulation factor XIII level | Spinal artery embolism | Activated protein C resistance test positive | Renal-limited thrombotic microangiopathy |
| Coagulation factor XIII level abnormal | Subclavian vein thrombosis | Hyperprothrombinaemia | ISTH score for disseminated intravascular coagulation |
| Coagulation factor XIII level decreased | Anti factor VIII antibody test | Tumour thrombosis | Fibrinogen degradation products increased |
| Coagulation factor XIII level increased | Abnormal clotting factor | Femoral artery embolism | Postoperative hypercoagulability |
| Coagulation time abnormal | Paget-Schroetter syndrome | Acquired protein S deficiency | Thrombin generation assay |
| Coagulation time normal | Thrombosis in device | Platelet-large cell ratio | Acquired factor V deficiency |
| Haemophilia | Protein C | Platelet-large cell ratio increased | Anti-platelet factor 4 antibody positive |
| Factor VIII deficiency | Protein S | Platelet-large cell ratio decreased | Thrombosis with thrombocytopenia syndrome |
| Factor XI deficiency | Mean platelet volume | Device embolisation | Anti-platelet factor 4 antibody test |
| Disseminated intravascular coagulation | Antithrombin III | Venous recanalisation | Anti-platelet factor 4 antibody negative |
| Coronary artery embolism | Platelet aggregation inhibition | Splenic embolism | Ophthalmic artery occlusion |
| Coronary artery thrombosis | Factor V Leiden mutation | Sticky platelet syndrome | Hypergammaglobulinaemic purpura of Waldenstrom |
| International normalised ratio decreased | Platelet aggregation test | Von Willebrand's factor antigen decreased | Septic cerebral embolism |
| Venous thrombosis in pregnancy | Heparin-induced thrombocytopenia test | Von Willebrand's factor antigen normal | Factor VIII activity test |
| Deep vein thrombosis | Postoperative thrombosis | Von Willebrand's factor activity abnormal | Aneurysm thrombosis |
| Postpartum venous thrombosis | Platelet distribution width | Von Willebrand's factor activity normal | Peripheral vein thrombosis |
| Purpura | Antithrombin III increased | Von Willebrand's factor activity test | Sigmoid sinus thrombosis |
| Platelet destruction increased | Protein S decreased | Von Willebrand's factor activity decreased | Thrombomodulin increased |
| Disseminated intravascular coagulation in newborn | Acquired dysfibrinogenaemia | Von Willebrand's factor activity increased | Heparin cofactor II deficiency |
| Ecchymosis | Congenital dysfibrinogenaemia | Von Willebrand's factor antigen test | Radiation thrombocytopenia |
| Embolic stroke | Hyperfibrinogenaemia | Von Willebrand's factor antigen abnormal | Ophthalmic vascular thrombosis |
| Embolism | Graft thrombosis | Venous intravasation | Dysmegakaryopoiesis |
| Iliac artery embolism | Hyperhomocysteinaemia | Cutaneovisceral angiomatosis with thrombocytopenia | Factor VIII activity increased |
| Embolism venous | Protein S deficiency | Pulmonary oil microembolism | Factor VIII activity normal |
| Renal vein embolism | Protein C deficiency | Henoch-Schonlein purpura nephritis | Factor VIII activity abnormal |
| Vena cava embolism | Thrombin time abnormal | Acquired Von Willebrand's disease | Factor VIII activity decreased |
| Septic embolus | Protein S increased | Platelet distribution width abnormal | Aortic aneurysm thrombosis |
| Mesenteric artery embolism | Protein S abnormal | Intrapericardial thrombosis | Coagulation factor level abnormal |
| Hypoprothrombinaemia | Protein S normal | Methylenetetrahydrofolate reductase polymorphism | Mesenteric vein embolism |
| Factor II deficiency | Haemangioma-thrombocytopenia syndrome | Thrombopoietin level abnormal | Spermatic vein thrombosis |
| Factor VII deficiency | Factor IX inhibition | Metastatic pulmonary embolism |  |
| Von Willebrand's disease | Factor XII deficiency | Vascular graft thrombosis |  |
| Factor XIII deficiency | Cerebrospinal thrombotic tamponade | Megakaryocyte destruction increased |  |
| Fibrin degradation products | Factor III deficiency | Methylenetetrahydrofolate reductase deficiency |  |

**Supplementary Table 3. Search terms for malignancy indications in MedDRA 25.1.**

| Acute lymphocytic leukaemia | Gastrooesophageal cancer | Diffuse large B-cell lymphoma | Colon cancer |
| --- | --- | --- | --- |
| Transitional cell carcinoma | Plasma cell myeloma | Nephroblastoma | Non-Hodgkin's lymphoma |
| Natural killer-cell lymphoblastic lymphoma | Fallopian tube cancer | Breast cancer | Clear cell sarcoma of soft tissue |
| Invasive ductal breast carcinoma | Leiomyosarcoma | Desmoplastic small round cell tumour | Acute myeloid leukaemia |
| Acute promyelocytic leukaemia | Peripheral T-cell lymphoma unspecified stage III | Adenocarcinoma gastric | Breast cancer stage III |
| Precursor T-lymphoblastic lymphoma/leukaemia | Acute leukaemia | Gastric cancer | B precursor type acute leukaemia |
| Hepatic cancer | Hodgkin's disease | Mantle cell lymphoma | Anaplastic large cell lymphoma T- and null-cell types |
| Acute monocytic leukaemia | Anaplastic large-cell lymphoma | Ovarian granulosa cell tumour | Endometrial cancer |
| Sarcoma | Ovarian cancer | Hepatocellular carcinoma | Chronic myeloid leukaemia |
| Breast cancer female | Myelodysplastic syndrome | Hepatic cancer stage III | Triple negative breast cancer |
| Breast cancer stage IV | Lymphoma | Ovarian epithelial cancer | Chronic lymphocytic leukaemia |
| Adenocarcinoma | Malignant peritoneal neoplasm | Gastrointestinal carcinoma | Small cell lung cancer |
| Lymphocytic lymphoma | Anal cancer | Neuroblastoma | Mesothelioma |
| Diffuse large B-cell lymphoma stage III | Oesophageal carcinoma | Plasma cell leukaemia | Endometrial adenocarcinoma |
| Breast cancer stage II | Sarcomatoid mesothelioma | Lung neoplasm malignant | Diffuse large B-cell lymphoma stage IV |
| Gastric cancer stage IV | Soft tissue sarcoma | Neoplasm malignant | Osteosarcoma |
| Burkitt's lymphoma | Adrenocortical carcinoma | Primary mediastinal large B-cell lymphoma | Ewing's sarcoma |
| Ovarian cancer stage IV | Renal cell carcinoma | Chronic myeloid leukaemia transformation | Inflammatory carcinoma of the breast |
| Oesophageal squamous cell carcinoma | Mantle cell lymphoma stage IV | Liposarcoma | Oesophageal adenocarcinoma |
| Malignant neoplasm of thymus | Non-Hodgkin's lymphoma unspecified histology aggressive | Central nervous system lymphoma | Angioimmunoblastic T-cell lymphoma stage III |
| Nodular lymphocyte predominant Hodgkin lymphoma | Angioimmunoblastic T-cell lymphoma | Burkitt's lymphoma stage IV | Plasmablastic lymphoma |
| Adenocarcinoma pancreas | Uterine leiomyosarcoma | Paget's disease of penis | Post transplant lymphoproliferative disorder |
| Primary effusion lymphoma | Dedifferentiated liposarcoma | Peripheral T-cell lymphoma unspecified | Burkitt's leukaemia |
| Hormone receptor positive HER2 negative breast cancer | Follicle centre lymphoma, follicular grade I, II, III | Prostate cancer | Neonatal neuroblastoma |
| Follicular lymphoma | Hodgkin's disease mixed cellularity stage IV | Invasive breast carcinoma | Philadelphia positive acute lymphocytic leukaemia |
| Non-renal cell carcinoma of kidney | Acute biphenotypic leukaemia | Uterine cancer | Precursor B-lymphoblastic lymphoma |
| Acute myeloid leukaemia refractory | Blastic plasmacytoid dendritic cell neoplasia | Adult T-cell lymphoma/leukaemia | Precursor T-lymphoblastic leukaemia acute |
| Primary mediastinal large B-cell lymphoma stage II | Acute myelomonocytic leukaemia | Breast implant-associated anaplastic large cell lymphoma | Synovial sarcoma |
| Kaposi's sarcoma | B-cell lymphoma refractory | Non-small cell lung cancer | High-grade B-cell lymphoma |
| High grade B-cell lymphoma Burkitt-like lymphoma stage IV | High grade B-cell lymphoma Burkitt-like lymphoma | Hodgkin's disease lymphocyte depletion type stage IV | Pleuropulmonary blastoma |
| Adrenal gland cancer | Hepatic angiosarcoma | Ovarian clear cell carcinoma | Gestational trophoblastic tumour |
| Hodgkin's disease stage IV | Clear cell renal cell carcinoma | Metastases to bone | Metastases to liver |
| Clear cell sarcoma of the kidney | Colorectal cancer | Small cell lung cancer extensive stage | Ovarian cancer stage III |
| Epithelioid sarcoma | Fibrosarcoma | Hodgkin's disease nodular sclerosis stage III | Sarcoma uterus |
| Diffuse large B-cell lymphoma stage II | Enteropathy-associated T-cell lymphoma | Diffuse large B-cell lymphoma stage I | Plasma cell myeloma refractory |
| Marginal zone lymphoma | Adenocarcinoma of colon | Lymphoma transformation | Rhabdomyosarcoma |
| Angiocentric lymphoma | Genital neoplasm malignant female | Ovarian cancer stage I | Myxofibrosarcoma |
| Cardiac neoplasm malignant | Chloroma | Follicular dendritic cell sarcoma | Breast cancer male |
| Serous cystadenocarcinoma ovary | T-cell lymphoma unclassifiable | Alveolar rhabdomyosarcoma | Malignant lymphoid neoplasm |
| Brain neoplasm malignant | Bone sarcoma | Pancreatic carcinoma | Mantle cell lymphoma refractory |
| Hepatosplenic T-cell lymphoma | Choriocarcinoma | Angiosarcoma | Thyroid cancer |
| Lymphocytic leukaemia |  |  |  |

**Supplementary Table 4. Adverse drug reaction listing table used for calculating ROR.**

| 2x2 contingency table for investigating associations between Anthracycline and the coagulation adverse events in patients with cancers. | | |
| --- | --- | --- |
| **Exposure** | **Coagulation Adverse Events** | **Other Adverse Events** |
| With Anthracycline | a | b |
| Without Anthracycline | c | d |

(a) represents cases with concurrent anthracycline use and coagulation-related adverse events; (b) represents anthracycline-treated cases without coagulation events; (c) represents non-anthracycline cases with coagulation events; (d) represents cases without anthracycline exposure or coagulation events. This contingency table formed the foundation for subsequent ROR analyses.

Abbreviations: ROR, reporting odds ratio.

**Supplementary Table 5. Sensitivity analysis: disproportionality signals (ROR with 95% CI) for clinically overt thrombotic HC-AEs across FAERS and VigiBase databases.**

| **Preferred Term** | **VigiBase** | | | | | | | | | **FAERS** | | | | | | | | |
| --- | --- | --- | --- | --- | --- | --- | --- | --- | --- | --- | --- | --- | --- | --- | --- | --- | --- | --- |
|  | **ROR** | **95% CI Upper** | **95% CI Lower** | **PRR** | **PRR_Upper** | **PRR_Lower** | **IC** | **IC025** | **n** | **ROR** | **95% CI Upper** | **95% CI Lower** | **PRR** | **PRR_Upper** | **PRR_Lower** | **IC** | **IC025** | **n** |
| **Atrial thrombosis** | 2.51 | 6.22 | 1.01 | 2.51 | 6.22 | 1.01 | 1.10 | -0.47 | 5 | 2.23 | 3.88 | 1.28 | 2.23 | 3.88 | 1.28 | 1.01 | 0.11 | 14 |
| **Cardiac ventricular thrombosis** | 4.32 | 9.00 | 2.07 | 4.32 | 9.00 | 2.07 | 1.75 | 0.54 | 8 | 5.59 | 7.90 | 3.95 | 5.59 | 7.90 | 3.95 | 2.11 | 1.60 | 42 |
| **Catheter site thrombosis** | 5.09 | 17.07 | 1.52 | 5.09 | 17.07 | 1.52 | 1.60 | -0.47 | 3 | 5.16 | 10.09 | 2.64 | 5.16 | 10.09 | 2.64 | 1.89 | 0.87 | 11 |
| **Cerebral artery embolism** | 2.85 | 7.89 | 1.03 | 2.85 | 7.89 | 1.03 | 1.19 | -0.58 | 4 | 2.58 | 5.01 | 1.33 | 2.58 | 5.01 | 1.33 | 1.16 | 0.08 | 10 |
| **Cerebral venous sinus thrombosis** | 4.85 | 8.47 | 2.77 | 4.84 | 8.47 | 2.77 | 1.97 | 1.07 | 14 | 5.35 | 8.22 | 3.48 | 5.35 | 8.22 | 3.48 | 2.03 | 1.39 | 27 |
| **Cerebral venous thrombosis** | 4.46 | 8.34 | 2.38 | 4.45 | 8.34 | 2.38 | 1.84 | 0.82 | 11 | 7.45 | 9.73 | 5.70 | 7.44 | 9.73 | 5.70 | 2.43 | 2.05 | 76 |
| **Deep vein thrombosis** | 1.27 | 1.44 | 1.12 | 1.27 | 1.44 | 1.12 | 0.33 | 0.13 | 265 | 1.41 | 1.53 | 1.30 | 1.41 | 1.52 | 1.30 | 0.46 | 0.33 | 621 |
| **Device related thrombosis** | 3.56 | 6.30 | 2.02 | 3.56 | 6.30 | 2.02 | 1.62 | 0.68 | 13 | 6.30 | 8.48 | 4.68 | 6.30 | 8.47 | 4.68 | 2.25 | 1.82 | 59 |
| **Disseminated intravascular coagulation** | 2.44 | 2.98 | 2.01 | 2.44 | 2.97 | 2.01 | 1.22 | 0.90 | 106 | 1.84 | 2.11 | 1.60 | 1.84 | 2.10 | 1.60 | 0.81 | 0.59 | 229 |
| **Embolism** | 2.62 | 3.22 | 2.13 | 2.62 | 3.22 | 2.13 | 1.31 | 0.98 | 96 | 2.21 | 2.55 | 1.91 | 2.20 | 2.55 | 1.91 | 1.05 | 0.82 | 204 |
| **Embolism venous** | 3.03 | 4.43 | 2.08 | 3.03 | 4.43 | 2.07 | 1.48 | 0.86 | 29 | 3.67 | 4.61 | 2.92 | 3.66 | 4.61 | 2.91 | 1.66 | 1.31 | 88 |
| **Intracardiac thrombus** | 3.29 | 5.41 | 2.00 | 3.29 | 5.41 | 2.00 | 1.55 | 0.73 | 17 | 7.77 | 9.94 | 6.07 | 7.76 | 9.93 | 6.07 | 2.48 | 2.13 | 91 |
| **Jugular vein thrombosis** | 2.20 | 3.85 | 1.25 | 2.20 | 3.84 | 1.25 | 1.03 | 0.09 | 13 | 3.94 | 5.20 | 2.99 | 3.94 | 5.20 | 2.99 | 1.74 | 1.32 | 61 |
| **Pulmonary embolism** | 1.21 | 1.35 | 1.08 | 1.21 | 1.35 | 1.08 | 0.26 | 0.07 | 303 | 1.37 | 1.47 | 1.27 | 1.36 | 1.46 | 1.27 | 0.42 | 0.31 | 821 |
| **Pulmonary infarction** | 5.22 | 9.56 | 2.85 | 5.22 | 9.56 | 2.85 | 2.03 | 1.05 | 12 | 3.34 | 5.17 | 2.16 | 3.34 | 5.17 | 2.16 | 1.51 | 0.83 | 24 |
| **Pulmonary veno-occlusive disease** | 10.48 | 21.22 | 5.18 | 10.48 | 21.21 | 5.18 | 2.63 | 1.55 | 10 | 5.54 | 8.85 | 3.47 | 5.54 | 8.84 | 3.47 | 2.06 | 1.36 | 23 |
| **Renal-limited thrombotic microangiopathy** | 5.94 | 17.12 | 2.06 | 5.94 | 17.12 | 2.06 | 1.83 | 0.07 | 4 | 5.51 | 9.49 | 3.20 | 5.51 | 9.49 | 3.20 | 2.02 | 1.20 | 17 |
| **Septic embolus** | 7.80 | 17.67 | 3.44 | 7.80 | 17.66 | 3.44 | 2.26 | 0.96 | 7 | 3.74 | 6.80 | 2.06 | 3.74 | 6.79 | 2.06 | 1.59 | 0.65 | 13 |
| **Subclavian vein thrombosis** | 4.46 | 7.93 | 2.50 | 4.45 | 7.93 | 2.50 | 1.87 | 0.93 | 13 | 8.39 | 11.53 | 6.11 | 8.39 | 11.52 | 6.11 | 2.53 | 2.08 | 56 |
| **Superior sagittal sinus thrombosis** | 4.46 | 11.29 | 1.76 | 4.45 | 11.29 | 1.76 | 1.67 | 0.11 | 5 | 5.06 | 8.41 | 3.04 | 5.05 | 8.41 | 3.04 | 1.94 | 1.17 | 19 |
| **Thrombophlebitis** | 4.86 | 6.66 | 3.55 | 4.85 | 6.65 | 3.54 | 2.08 | 1.58 | 44 | 2.18 | 2.95 | 1.60 | 2.17 | 2.95 | 1.60 | 1.02 | 0.53 | 46 |
| **Thrombotic microangiopathy** | 1.58 | 2.11 | 1.18 | 1.58 | 2.11 | 1.18 | 0.63 | 0.14 | 47 | 1.59 | 1.89 | 1.34 | 1.59 | 1.89 | 1.34 | 0.62 | 0.35 | 143 |
| **Transverse sinus thrombosis** | 10.97 | 33.63 | 3.58 | 10.97 | 33.63 | 3.58 | 2.22 | 0.46 | 4 | 5.94 | 13.98 | 2.53 | 5.94 | 13.97 | 2.53 | 1.92 | 0.61 | 7 |
| **Venous thrombosis** | 3.38 | 4.75 | 2.40 | 3.38 | 4.75 | 2.40 | 1.62 | 1.07 | 36 | 7.50 | 8.79 | 6.40 | 7.49 | 8.77 | 6.39 | 2.46 | 2.24 | 218 |
| **Venous thrombosis limb** | 1.85 | 3.06 | 1.12 | 1.85 | 3.06 | 1.12 | 0.82 | -0.02 | 16 | 2.33 | 3.20 | 1.70 | 2.33 | 3.20 | 1.70 | 1.10 | 0.60 | 44 |

Abbreviations: 95% CI Lower, 95% Confidence Interval Lower Limit; 95% CI Upper, 95% Confidence Interval Upper Limit; FAERS, Food and Drug Administration Adverse Event Reporting System; IC, Information Component; IC025, Information Component 2.5% Lower Quantile; PRR, Proportional Reporting Ratio; PRR_Lower, Lower Limit of the 95% Confidence Interval for Proportional Reporting Ratio; PRR_Upper, Upper Limit of the 95% Confidence Interval for Proportional Reporting Ratio; PT, preferred term; ROR, reporting odds ratio; VigiBase, WHO Global Database of Individual Case Safety Reports.

**Supplementary Table 6. Sensitivity analysis of time-to-onset (TTO) using only reports with completely precise dates.**

| **Variables** | **Subgroups** | **Primary Analysis (Imputed + Precise)**  **Median TTO [IQR], days** | **Sensitivity Analysis (Precise Only)**  **Median TTO [IQR], days** | **P-value**  **(Sensitivity)** |
| --- | --- | --- | --- | --- |
| **Exposure** | With Anthracyclines | 32.5 [13.0–85.3] | 29.0 [13.0–76.0] | < 0.001 |
|  | Without Anthracyclines | 48.0 [14.0–123.0] | 41.0 [13.0–106.0] |  |
| **Sex** | Male | 39.0 [16.0–92.5] | 38.0 [16.0–85.5] | 0.056 |
|  | Female | 40.0 [13.8–84.3] | 30.0 [13.0–78.0] |  |
| **Age** | ≤39 years | 21.5 [10.0–56.8] | 21.0 [10.0–53.0] | < 0.001 |
|  | 40–59 years | 57.0 [16.0–109.0] | 45.5 [12.8–101.5] |  |
|  | ≥60 years | 47.0 [16.0–84.0] | 40.0 [16.0–80.0] |  |
| **Drug Types** | Daunorubicin | 16.0 [5.0–29.5] | 15.0 [5.0–26.0] | < 0.001 |
|  | Idarubicin | 25.0 [7.5–30.5] | 23.0 [5.0–30.0] |  |
|  | Aclarubicin | 38.0 [18.3–68.5] | 38.0 [18.3–68.5] |  |
|  | Doxorubicin | 38.0 [14.0–91.0] | 33.5 [13.8–88.0] |  |
|  | Epirubicin | 64.5 [26.5–88.5] | 53.0 [21.0–84.5] |  |

**Supplementary Figure 1. Comparative landscape of HC-AEs signals across different anthracyclines in FAERS and VigiBase databases.**

This bubble plot illustrates the disproportionality analysis of HC-AEs associated with seven anthracycline agents (Doxorubicin, Daunorubicin, Epirubicin, Idarubicin, Aclarubicin, Amrubicin, and Pirarubicin) utilizing data from FAERS and VigiBase. The y-axis lists specific HC-AEs classified by MedDRA PTs, ranging from common venous thromboembolism to specific entities like cerebral venous thrombosis and thrombophlebitis. The x-axis stratifies the analysis by specific drug and data source. Bubble size is proportional to the absolute number of reported cases, reflecting the volume of safety data. Color intensity represents the signal strength measured by the ROR, with a gradient ranging from low signal (light red) to high signal intensity (deep red, ROR ≥ 30) . The visualization highlights drug-specific heterogeneity in HC-AEs profiles.

Abbreviations: FAERS, Food and Drug Administration Adverse Event Reporting System; HC-AEs, hypercoagulable adverse events; PT, preferred term; ROR, reporting odds ratio; VigiBase, WHO Global Database of Individual Case Safety Reports.

**Supplementary Figure 2. Stratification of HC-AEs signal intensity by underlying malignancy type.**

This figure displays a subgroup analysis of anthracycline-associated HC-AEs, stratified by the primary indication for treatment. The x-axis categorizes reports into distinct tumor types, including Hematological Malignancies, Breast Cancer, Sarcoma and Bone Tumors, Gastrointestinal Malignancies, and others, compared against the "Overall" population. The y-axis enumerates specific HC-AEs phenotypes. Similar to Supplementary Figure 1, the size of each bubble corresponds to the case count, while the color gradient signifies the magnitude of the ROR. This analysis reveals disease-specific patterns in HC-AEs, distinguishing events driven by the underlying malignancy (e.g., hypercoagulability in hematological malignancies) from those universally observed across indications.

Abbreviations: HC-AEs, hypercoagulable adverse events; ROR, reporting odds ratio.
